# Formoterol attenuates increased oxidative stress and myosin protein loss in respiratory and limb muscles of cancer cachectic rats

**DOI:** 10.7717/peerj.4109

**Published:** 2017-12-13

**Authors:** Anna Salazar-Degracia, Sílvia Busquets, Josep M. Argilés, Francisco J. López-Soriano, Esther Barreiro

**Affiliations:** 1 Pulmonology Department-Muscle Wasting and Cachexia in Chronic Respiratory Diseases and Lung Cancer Research Group, Health and Experimental Sciences Department (CEXS), IMIM-Hospital del Mar, Parc de Salut Mar, Universitat Pompeu Fabra (UPF), Barcelona Biomedical Research Park (PRBB), Barcelona, Spain; 2 Cancer Research Group, Departament de Bioquímica i Biomedicina Molecular, Facultat de Biologia, Universitat de Barcelona, Universitat de Barcelona, Barcelona, Spain; 3 Institut de Biomedicina de la Universitat de Barcelona (IBUB), Barcelona, Spain; 4 Centro de Investigación en Red de Enfermedades Respiratorias (CIBERES), Instituto de Salud Carlos III (ISCIII), Barcelona, Spain

**Keywords:** Experimental cancer-induced cachexia, Diaphragm and gastrocnemius, Formoterol treatment, Redox balance, Contractile proteins

## Abstract

Muscle mass loss and wasting are characteristic features of patients with chronic conditions including cancer. Therapeutic options are still scarce. We hypothesized that cachexia-induced muscle oxidative stress may be attenuated in response to treatment with beta_2_-adrenoceptor-selective agonist formoterol in rats. In diaphragm and gastrocnemius of tumor-bearing rats (108 AH-130 Yoshida ascites hepatoma cells inoculated intraperitoneally) with and without treatment with formoterol (0.3 mg/kg body weight/day for seven days, daily subcutaneous injection), redox balance (protein oxidation and nitration and antioxidants) and muscle proteins (1-dimensional immunoblots), carbonylated proteins (2-dimensional immunoblots), inflammatory cells (immunohistochemistry), and mitochondrial respiratory chain (MRC) complex activities were explored. In the gastrocnemius, but not the diaphragm, of cancer cachectic rats compared to the controls, protein oxidation and nitration levels were increased, several functional and structural proteins were carbonylated, and in both study muscles, myosin content was reduced, inflammatory cell counts were greater, while no significant differences were seen in MRC complex activities (I, II, and IV). Treatment of cachectic rats with formoterol attenuated all the events in both respiratory and limb muscles. In this *in vivo* model of cancer-cachectic rats, the diaphragm is more resistant to oxidative stress. Formoterol treatment attenuated the rise in oxidative stress in the limb muscles, inflammatory cell infiltration, and the loss of myosin content seen in both study muscles, whereas no effects were observed in the MRC complex activities. These findings have therapeutic implications as they demonstrate beneficial effects of the beta_2_ agonist through decreased protein oxidation and inflammation in cachectic muscles, especially the gastrocnemius.

## Introduction

Muscle mass loss and wasting are characteristic features of patients with chronic conditions such as chronic heart failure, diabetes, renal failure, chronic obstructive pulmonary disease (COPD), cancer, and critical illness (Alvarez et al., 2016; Barreiro et al., 2015; Barreiro, 2017; Diaz, Ospina-Tascon & Salazar, 2014; Evans et al., 2008; Fearon et al., 2011; Villar et al., 2016; Von & Anker, 2010) Cachexia is characterized by severe body weight and muscle loss together with alterations in metabolic parameters. Patients with cachexia experience severe muscle weakness, which has been consistently demonstrated to predict morbidity and mortality (Barreiro et al., 2015; Evans et al., 2008; Fearon et al., 2011; Mador, Bozkanat & Kufel, 2003; Marquis et al., 2002; Patel et al., 2014; Seymour et al., 2010; Swallow et al., 2007). Importantly, muscle wasting also negatively impacts on the patients’ daily physical activities, thus severely limiting their quality of life.

Several biological mechanisms have been proposed to participate in the pathophysiology of muscle wasting associated with chronic diseases including cancer such as oxidative stress, systemic inflammation, epigenetics, metabolic derangements, sarcomere disruptions, contractile protein loss, and enhanced proteolysis (Barreiro et al., 2003; Barreiro et al., 2008; Barreiro et al., 2010; Barreiro et al., 2011; Barreiro, 2017; Chacon-Cabrera et al., 2015; Chacon-Cabrera et al., 2016; Chacon-Cabrera, Gea & Barreiro, 2016; Fermoselle et al., 2012; Fermoselle et al., 2013; Fontes-Oliveira et al., 2013; Fontes-Oliveira et al., 2014; Marin-Corral et al., 2009; Marin-Corral et al., 2010; Puig-Vilanova et al., 2014c; Puig-Vilanova et al., 2014a; Puig-Vilanova et al., 2014b; Puig-Vilanova et al., 2015; Chacon-Cabrera et al., 2014). Oxidative stress, defined as the imbalance between oxidants and antioxidants in favor of the former, has been implicated in the pathogenesis of several conditions including chronic disease-associated muscle wasting and cachexia (Barreiro et al., 2003; Barreiro et al., 2008; Barreiro et al., 2010; Barreiro et al., 2011; Barreiro, 2017; Fermoselle et al., 2012; Marin-Corral et al., 2009; Marin-Corral et al., 2010; Puig-Vilanova et al., 2014c). Proteins, DNA, and lipids are the main cellular targets for the action of those oxidants that escape the tissue antioxidant capacity, thus leading to deleterious effects on cells including increased susceptibility to proteolytic degradation. Reaction of oxidants with specific amino acids leads to the formation of reactive carbonylation derivatives (aldehydes and ketones) that have been extensively analyzed as a reliable marker of protein oxidation (Stadtman & Levine, 2003; Barreiro, 2016). Reactive carbonyl groups may also be the result of Michael-addition reactions of other residues with α, β-unsaturated aldehydes (e.g., malondialdehyde, MDA) as by-products of fatty acid oxidation (Friguet, Stadtman & Szweda, 1994). Interestingly, the highly reactive species peroxynitrite, formed by the near-diffusion limited reaction between nitric oxide and superoxide anion, may also oxidize proteins or nitrate tyrosine residues leading to nitrosative stress (Beckman & Koppenol, 1996). A rise in all these oxidative stress markers has been demonstrated in the peripheral muscles of patients with muscle wasting (Barreiro et al., 2003; Barreiro et al., 2008; Barreiro et al., 2010; Fermoselle et al., 2012; Marin-Corral et al., 2009; Puig-Vilanova et al., 2014c) and animals with experimental cancer-induced cachexia (Chacon-Cabrera et al., 2014; Fermoselle, Sanchez & Barreiro, 2011; Fermoselle et al., 2013; Marin-Corral et al., 2010; Salazar-Degracia et al., 2016). Whether increased oxidative stress may also take place in the respiratory muscles in cancer cachexia models remains to be fully elucidated.

In clinical settings, availability of effective therapeutic options for cachexia is still scarce. Significant skeletal muscle growth can be achieved in response to treatment with beta-adrenoceptor agonists when administered at higher doses than those normally used for the treatment of airways diseases, namely asthma and COPD. Despite that skeletal muscles possess all three subtypes of beta-adrenoceptors, the proportions of beta_2_ receptors are significantly more abundant than those of beta_1_- and beta_3_-adrenoceptors (Williams, Caron & Daniel, 1984). Canonical beta-agonist signaling is characterized by initiation of downstream signaling by a heterotrimeric G-protein that couples to adenylate cyclase, resulting in the conversion of adenosine triphosphate (ATP) to cyclic adenosine monophosphate (cAMP), which in turn activates protein kinase A (Ryall & Lynch, 2008). The beneficial effects of the activation of this pathway on muscles involve a decrease in protein degradation and an increase in protein anabolism or a combination of both (Ryall & Lynch, 2008). In keeping with, the long-acting beta_2_ agonist formoterol induced beneficial effects in muscle mass loss and function (Busquets et al., 2004) and physical activity (Busquets et al., 2011) of rats with cancer cachexia by attenuating several biological events and processes such as myostatin (Busquets et al., 2012; Toledo et al., 2016), and proteasome activity (Busquets et al., 2004). Whether additional effects may be expected on other biological factors such as muscle oxidative stress, metabolism and inflammation needs to be further investigated.

On this basis, we hypothesized that cachexia-induced muscle oxidative stress may be attenuated in response to treatment of the animals with the beta_2_ agonist formoterol. In the present study, the analysis of the diaphragm muscle, which must remain continuously active, has also been included as most of the investigations published so far have mainly focused on the evaluation of the peripheral muscles. Such an approach also enabled us to identify whether the activity of the muscle influenced the profile of biological events.

Taking all this into consideration, in the study, we focused on the analysis of diaphragm and gastrocnemius of cachectic rats bearing the Yoshida ascites hepatoma (Busquets et al., 2004; Busquets et al., 2011; Busquets et al., 2012; Fontes-Oliveira et al., 2013; Fontes-Oliveira et al., 2014; Toledo et al., 2011; Toledo et al., 2016), and the following biological events were analyzed in response to treatment with formoterol: (1) oxidative stress markers, antioxidants, and activity of mitochondrial complexes of the respiratory chain, (2) inflammatory cells, and (3) levels of intramuscular specific muscle proteins known to be oxidized in muscles.

## Materials and Methods

(Detailed information on all methodologies is described in the online Supplementary Material).

### Animal experiments

#### Experimental design

Male Wistar rats (five weeks, 130–165 grams, Interfauna, Barcelona, Spain) were used for the purpose of the investigation. Animals were randomly subdivided into four groups (*N* = 10/group) and were studied for seven days: (1) non-cachexia controls, (2) non-cachexia controls treated with formoterol (non-cachexia control-F), (3) cancer-cachexia rats, and (4) cancer-cachexia rats treated with formoterol (cancer-cachexia-F). Cachexia was induced as a result of an intraperitoneal inoculum of 10^8^ AH-130 Yoshida ascites hepatoma cells, which were obtained from tumors in exponential growth as previously described (Busquets et al., 2004; Busquets et al., 2011; Busquets et al., 2012; Fontes-Oliveira et al., 2013; Fontes-Oliveira et al., 2014; Toledo et al., 2011; Toledo et al., 2016). AH-130 Yoshida ascites hepatoma is a well-validated model characterized by a rapid and progressive loss of body weight and muscle mass. As previously demonstrated (Lopez-Soriano, Argiles & Lopez-Soriano, 1997; Toledo et al., 2011), a moderate cachexia (8% of body weight loss) was already seen on day 4, while reaching 20–25% of body weight loss on day 7. For ethical reasons (large tumor sizes), seven days was established as the duration of the study.

Formoterol treatment was administered subcutaneously (0.3 mg/kg body weight/24 h, dissolved in physiological solution) six hours after inoculation of the tumor cells and was thereafter administered daily during seven consecutive days up until the sacrifice of the animals. Non-treated rats received the corresponding volume of physiological solution that was administered subcutaneously every day for seven days (Busquets et al., 2004; Busquets et al., 2011; Busquets et al., 2012; Fontes-Oliveira et al., 2013; Fontes-Oliveira et al., 2014; Toledo et al., 2011; Toledo et al., 2016). In this model of cancer cachexia, the most suited dose and duration of the formoterol treatment was established in previous investigations of our group (Busquets et al., 2004; Toledo et al., 2014). Importantly, administration of 0.3 mg/kg/24 h formoterol for seven days was the minimum dose that ensured a beneficial anti-cachectic effect in the rats, while no side-effects were induced on other organs including the heart (Busquets et al., 2004; Toledo et al., 2014).

All animal experiments were conducted at *Facultat de Biologia, Universitat de Barcelona (Barcelona)*. This was a controlled study designed in accordance with both the ethical standards on animal experimentation in our institution (EU 2010/63 CEE and *Real Decreto* 53/2013 BOE 34, Spain) and the Helsinki Convention for the Use and Care of Animals. Ethical approval was obtained by the institutional Animal Experimentation Ethics Committee (Reference number DAAM: 8315, *Universitat de Barcelona*).

### *In vivo* measurements in the animals

Food and water were administered *ad libitum* to the animals for the entire duration of the study. All the animals were maintained at a temperature of 22 ± 2 °C with a regular light-dark cycle (lights were on from 08:00 a.m. to 08:00 p.m.) and had free access to food and water. Body weight was determined in all animals on day 0 and prior to their sacrifice on day 7. Tumor weights were determined in all animals upon sacrifice. The percentage of body weight gain at the end of the period was calculated as follows: [(body weight on day 7 − tumor weight on day 7) − body weight on day 0]/ body weight on day 0 × 100 (Busquets et al., 2004; Busquets et al., 2011; Busquets et al., 2012; Fontes-Oliveira et al., 2013; Fontes-Oliveira et al., 2014; Toledo et al., 2011; Toledo et al., 2016).

### Sacrifice and sample collection

On day 7 after tumor transplantation, the animals were weighed and anesthetized through an intraperitoneal injection of 3:1 ketamine/xylazine mixture (Imalgene^®^ 1000; Rhone Merieux, France and Rompun^®^, Bayer AG, Leverkusen, Germany, respectively). In all animals, the pedal and blink reflexes were evaluated in order to verify total anesthesia depth. The diaphragm and gastrocnemius muscles were obtained from all the animals. In all samples, muscle specimens were immediately frozen in liquid nitrogen and subsequently stored at −80 °C. Frozen tissues were used to assess the expression of the target molecular markers (Busquets et al., 2004; Busquets et al., 2011; Busquets et al., 2012; Fontes-Oliveira et al., 2013; Fontes-Oliveira et al., 2014; Toledo et al., 2011; Toledo et al., 2016).

### Muscle biology analyses

All the muscle biological experiments were performed in the laboratory at *Hospital del Mar-* IMIM*-Universitat Pompeu Fabra* (Barcelona).

#### Detection of reactive carbonyls in muscle proteins

Changes in protein carbonylation in crude muscle homogenates were detected using the commercially available Oxyblot kit (Chemicon International Inc., Temecula, CA, USA). Carbonyl groups in the protein side chains were derivatized to 2,4-dinitrophenylhydrazone (DNP) by reaction with 2,4-dinitrophenylhydrazine (DNPH) according to the manufacturer’s instructions and previous methodologies (Barreiro et al., 2012; Marin-Corral et al., 2010).

#### Immunoblotting

The effects of ROS and RNS on muscle proteins were explored using previously published methodologies (Chacon-Cabrera et al., 2014; Chacon-Cabrera et al., 2015; Chacon-Cabrera et al., 2016; Chacon-Cabrera, Gea & Barreiro, 2016; Fermoselle et al., 2012; Puig-Vilanova et al., 2014c; Salazar-Degracia et al., 2016). Proteins were then separated by electrophoresis, transferred to polyvinylidene difluoride (PVDF) membranes, blocked with 5% non-fat milk or with 1% BSA, and incubated with the corresponding primary antibodies overnight for each of the target markers. Protein content of the different markers was identified using specific primary antibodies: anti-DNP moiety antibody (rabbit anti-DNP antibody from the Oxyblot kit), 4-hydroxy-2-nonenal (HNE)-protein adducts (anti-HNE-protein adducts antibody; Alpha Diagnostic International, San Antonio, TX, USA), malondialdehyde (MDA)-protein adducts (anti-MDA-protein adducts antibody; Academy Bio-Medical Company, Inc., Houston, TX, USA), 3-nitrotyrosine (anti-3-nitrotyrosine antibody, invitrogen, Eugene, Oregon, USA), superoxide dismutase (SOD)2 (anti-SOD2 antibody; Santa Cruz Biotechnology, Santa Cruz, CA, USA), SOD1 (anti-SOD1 antibody; Santa Cruz Biotechnology, Santa Cruz, CA, USA), catalase (anti-catalase antibody; Calbiochem, Darmstadt, Germany), MyHC-I (anti-MyHC-I antibody; Sigma-Aldrich, St. Louis, MO, USA), MyHC-II (anti-MyHC-II antibody; Abcam, Cambridge, UK), actin (anti-alpha-sarcomeric actin antibody, clone 5C5; Sigma Sigma-Aldrich, St. Louis, MO, USA), creatine kinase (anti-creatine kinase antibody; Santa Cruz Biotechnology, Santa Cruz, CA, USA), carbonic anhydrase-3 (anti-carbonic anhydrase-3 antibody; Santa Cruz Biotechnology, Santa Cruz, CA, USA), and glyceraldehyde-3-phosphate dehydrogenase (GAPDH, anti-GAPDH antibody, Santa Cruz). MyHC-I and MyHC-II isoforms were detected in the immunoblots from muscle homogenates, in which the myofibrilar compartment was isolated (Picard et al., 2011).

Values of total reactive carbonyl groups, total HNE- and MDA-protein adducts, and protein tyrosine nitration in a given sample were calculated by addition of the optical densities (arbitrary units) of individual protein bands in each case. Values of total MyHC were the sum of the optical densities obtained from the MyHC-I and MyHC-II immunoblots separately. Final optical densities obtained in each specific group of animals corresponded to the mean values of the different samples (lanes) of each of the study antigens. To validate equal protein loading across lanes, the glycolytic enzyme GAPDH was used as the protein loading control in all the immunoblots, except for MyHC-I and MyHC-II isoforms, in which Coomassie Blue staining was used (Figs. S1–S4 and S6–S13).

#### Identification of carbonylated muscle proteins using 2D electrophoresis

Following procedures previously published (Marin-Corral et al., 2009; Marin-Corral et al., 2010), two-dimensional gel electrophoresis was used to separate and identify the carbonylated proteins in the gastrocnemius muscles of all groups of rats.

#### Identification of carbonylated proteins using mass spectrometry (MS)

Identification of carbonylated proteins using mass spectrometry was conducted in the Proteomics Laboratory at *Universitat Pompeu Fabra (Barcelona)* following the quality criteria established by *ProteoRed* standards (*Instituto Nacional de Proteómica, Spain*) and procedures previously published (Marin-Corral et al., 2009).

#### Intramuscular cellular inflammation

In order to evaluate the presence of inflammatory cells in diaphragm and gastrocnemius muscles, immunohistochemical analyses were conducted in each muscle of all study groups following previously published methodologies and specific antibodies (Barreiro et al., 2011; Barreiro et al., 2010). On three-micrometer muscle paraffin-embedded sections, leukocytes (anti-CD45 antibody, clone 2B11 & PD7/26; Dako Cytomation Inc., Carpinteria, CA, USA) and macrophages (anti-CD68 antibody, clone PG-M1, Dako Cytomation Inc.) were identified following immunohistochemical procedures (Barreiro et al., 2011; Barreiro et al., 2010). Results corresponding to inflammatory cell counts were expressed as follows: the ratio of either leukocyte or macrophage numbers to total muscle section area and the ratio of both cell types to total muscle section area in both diaphragm and gastrocnemius muscles (Barreiro et al., 2011).

### Mitochondrial respiratory chain (MRC) complexes: enzyme activities in muscles

#### Homogenization procedures

Snap-frozen diaphragm and gastrocnemius muscles obtained from all rats were homogenized using a Homogenisator Potter S (Sartorius Stedim Biotech GmbH, Goettingen, Germany) following previously published methodologies (Fermoselle et al., 2013; Medja et al., 2009). The supernatants obtained from the second centrifugation were added to the first one, thus yielding the final sample supernatants. Protein concentrations were measured using the Bradford technique (Bradford, 1976).

#### Mitochondrial citrate synthase (CS) activity

The procedures employed to determine CS activity have also been previously reported (Fermoselle et al., 2013; Medja et al., 2009).

#### Mitochondrial complex I activity

All procedures employed in the current investigation have been published previously (Fermoselle et al., 2013; Medja et al., 2009).

#### Mitochondrial complex II activity

All these procedures have been previously reported (Fermoselle et al., 2013; Medja et al., 2009).

#### Mitochondrial complex IV activity

All procedures used for measuring this complex have been published previously (Fermoselle et al., 2013; Medja et al., 2009).

### Statistical analysis

The normality of the study variables was verified using the Shapiro–Wilk test. Results are presented as mean (standard deviation). The comparisons between the different study groups were analyzed using the one-way analysis of variance (ANOVA), in which Tukey’s post hoc analysis was used to adjust for multiple comparisons. The sample size chosen was based on previous studies (Busquets et al., 2004; Busquets et al., 2011; Busquets et al., 2012; Chacon-Cabrera et al., 2014; Chacon-Cabrera et al., 2015; Chacon-Cabrera et al., 2016; Chacon-Cabrera, Gea & Barreiro, 2016; Fermoselle, Sanchez & Barreiro, 2011; Fermoselle et al., 2013; Fontes-Oliveira et al., 2013; Fontes-Oliveira et al., 2014; Marin-Corral et al., 2010; Salazar-Degracia et al., 2016; Toledo et al., 2011; Toledo et al., 2016) and on assumptions of 80% power to detect an improvement of more than 20% in measured outcomes at a level of significance of *P* ≤ 0.05. All statistical analyses were performed using the Statistical Package for the Social Sciences (Portable SPSS, PASW statistics 18.0 version for Windows; SPSS Inc., Chicago, IL, USA).

## Results

### Physiological characteristics of the study animals

As shown in Table 1, at the end of the study period (day 7), cancer-cachexia rats exhibited a reduction in the variables final body weight and body weight gain compared to non-cachexia controls. In cancer-cachexia rats, formoterol treatment significantly improved body weight gain compared to the non-treated cachectic rats (Table 1). The weights of diaphragm and gastrocnemius muscles were significantly smaller in cancer-cachexia rats than in non-cachexia controls, and treatment with formoterol elicited a significant improvement in the weight of these muscles in the cachectic animals (Table 1).

**Table 1:** Physiological characteristics of rats in all the study groups.

	Non-cachexia controls	Non-cachexia control-formoterol	Cancer- cachexia	Cancer-cachexia- formoterol
	*N* = 9	*N* = 9	*N* = 10	*N* = 10
Initial body weight (g)	129.2 ± 6.4	122.31 ± 9.3	125.1 ± 11.4	124.5 ± 6.6
Tumor weight (g)	NA	NA	42.1 ± 8.8	38.8 ± 8.9
Final body weight (g)	166.6 ± 9.7	159.5 ± 9.3	116.7 ± 11.3^***^	127.2 ± 8.3
Body weight gain (%)	+29.0 ± 2.5	+30.6 ± 3.3	−6.6 ± 5.0^***^	+2.2 ± 5.5^§§§^
Diaphragm weight (mg/100 g IBW)	285.8 ± 33.9	325.6 ± 51.3	113.5 ± 24.2^***^	181.7 ± 106.3
Gastrocnemius weight (mg/100 g IBW)	663.3 ± 40.3	709.9 ± 60.3	544.6 ± 20.7^***^	632.4 ± 22.9^§§§^

**Notes.**

Variables are presented as mean ± standard deviation.

Abbreviations NA not applicable g gram mg milligram IBW initial body weight

Statistical significance:

*** *p* ≤ 0.001 between non-cachexia controls and cancer-cachexia rats.

§§§ *p* ≤ 0.001 between cancer-cachexia rats and cancer-cachexia rats treated with formoterol.

### Protein oxidation

**Figure 1:**
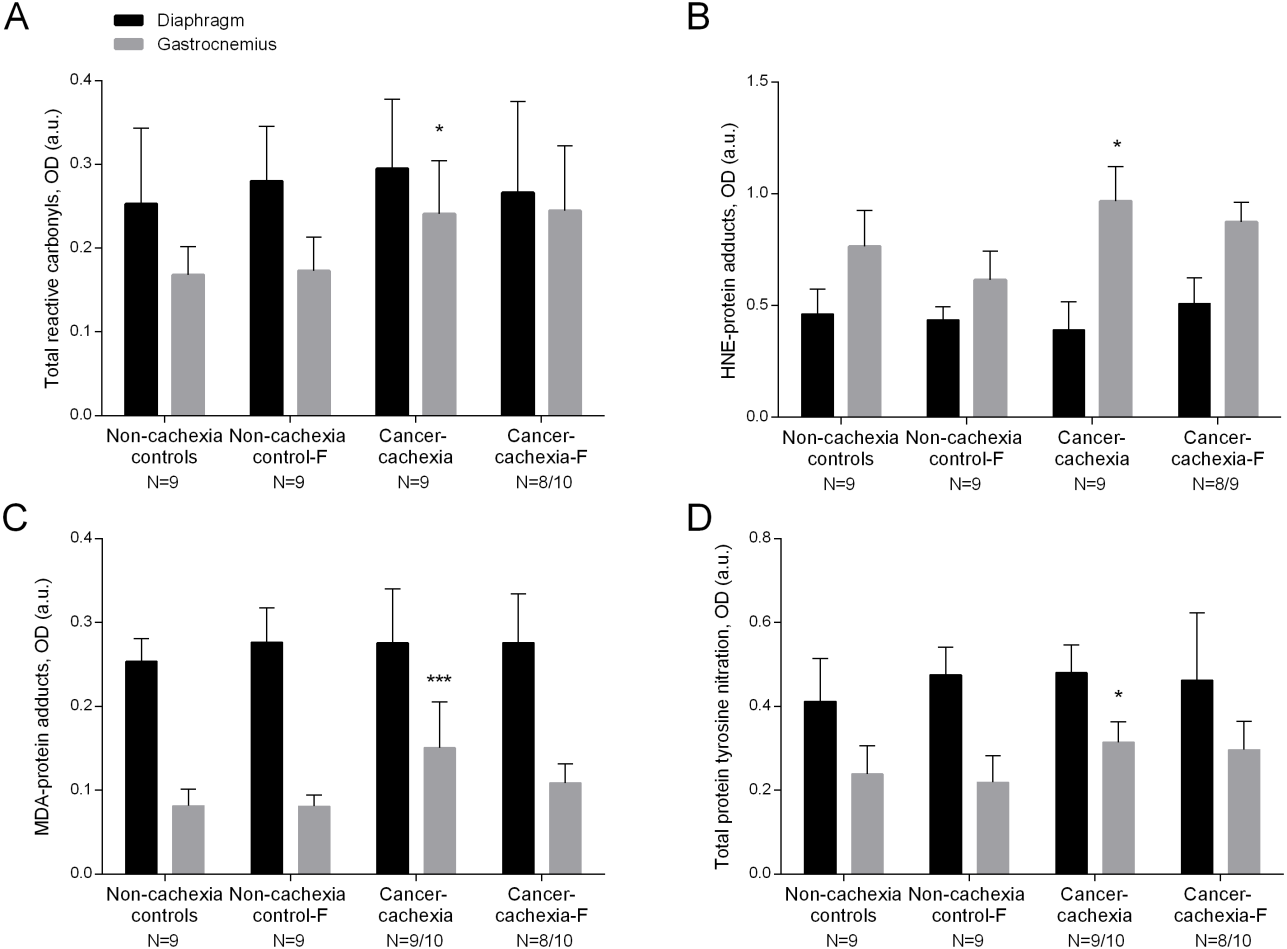
Levels of oxidative stress markers in diaphragm and gastrocnemius muscles. (A) Mean values and standard deviation of total reactive carbonyls in diaphragm (black bars) and gastrocnemius (grey bars) muscles as measured by optical densities in arbitrary units (OD, a.u.). Definition of abbreviations: F, formoterol. Statistical significance is represented as follow: * *p* ≤ 0.05 between non-cachexia controls and cancer-cachexia rats. (B) Mean values and standard deviation of HNE-protein adducts in diaphragm (black bars) and gastrocnemius (grey bars) muscles as measured by optical densities in arbitrary units (OD, a.u.). Definition of abbreviations: HNE, 4-hydroxy-2-nonenal; F, formoterol. Statistical significance is represented as follow: * *p* ≤ 0.05 between non-cachexia controls and cancer-cachexia rats. (C) Mean values and standard deviation of MDA-protein adducts in diaphragm (black bars) and gastrocnemius (grey bars) muscles as measured by optical densities in arbitrary units (OD, a.u.). Definition of abbreviations: MDA, malondialdehyde; F, formoterol. Statistical significance is represented as follow: *** *p* ≤ 0.001 between non-cachexia controls and cancer-cachexia rats; § *p* ≤ 0.05 between cancer-cachexia rats and cancer-cachexia rats treated with formoterol. (D) Mean values and standard deviation of total protein tyrosine nitration in diaphragm (black bars) and gastrocnemius (grey bars) muscles as measured by optical densities in arbitrary units (OD, a.u.). Definition of abbreviations: F, formoterol. Statistical significance is represented as follow: * *p* ≤ 0.05 between non-cachexia controls and cancer-cachexia rats.

Compared to non-cachexia controls, total reactive carbonyls, both HNE- and MDA- protein adducts, and total protein tyrosine nitration levels were increased only in the limb muscle of the cancer-cachexia rats (Figs. 1A–1D and Figs. S1–S4). Treatment with formoterol attenuated the rise in levels of total reactive carbonyls, HNE- and MDA-protein adducts, and protein tyrosine nitration in the gastrocnemius of the cancer-cachexia rats (Figs. 1A–1D and Figs. S1–S4). No significant differences were seen in any of these markers in respiratory or limb muscles between non-cachexia controls and non-cachexia control-formoterol animals (Figs. 1A–1D and Figs. S1–S4). Several glycolytic enzymes, albumin, actin and tropomyosin were shown to be carbonylated in gastrocnemius muscles of the study groups (Table 2 and Fig. S5).

**Table 2:** Identified carbonylated proteins in gastrocnemius muscles of all study groups.

Identified carbonylated proteins	Accession number	Mass (Da)	MASCOT scores
1. Isoforms of pyruvate kinase, cancer-cachexia and cancer-cachexia-formoterol groups	KPYM_RAT	58,294	152
2. Isoforms of βeta-enolase, all groups except for cancer-cachexia-formoterol group	ENOB_RAT	47,326	97
3. Isoforms of creatine kinase, all groups	KCRM_RAT	43,246	134
4. Isoforms of fructose biphosphate aldolase, all groups	ALDOA_RAT	39,783	107
5. Isoforms of glyceraldehyde-3-phosphate dehydrogenase, all groups	G3P_RAT	36,090	54
6. Isoforms of carbonic anhydrase-3, cancer-cachexia and cancer-cachexia-formoterol groups	CAH3_RAT	29,698	75
7. Albumin (non-muscle protein), all groups	ALBU_RAT	68,730	98
8. Adenosin-triphosphate synthase, all groups except for cancer-cachexia-formoterol group	ATPB_RAT	56,318	63
9. Actin, all groups except cancer-cachexia-formoterol group	ACTC_RAT	42,334	79
10. Tropomyosin, all groups	TPM1_RAT	32,837	64

**Notes.**

Database: SwissProt 56.0. Protein scores greater than 56 are significant (*p* ≤ 0.05).

Abbreviations Da daltons

### Antioxidants

**Figure 2:**
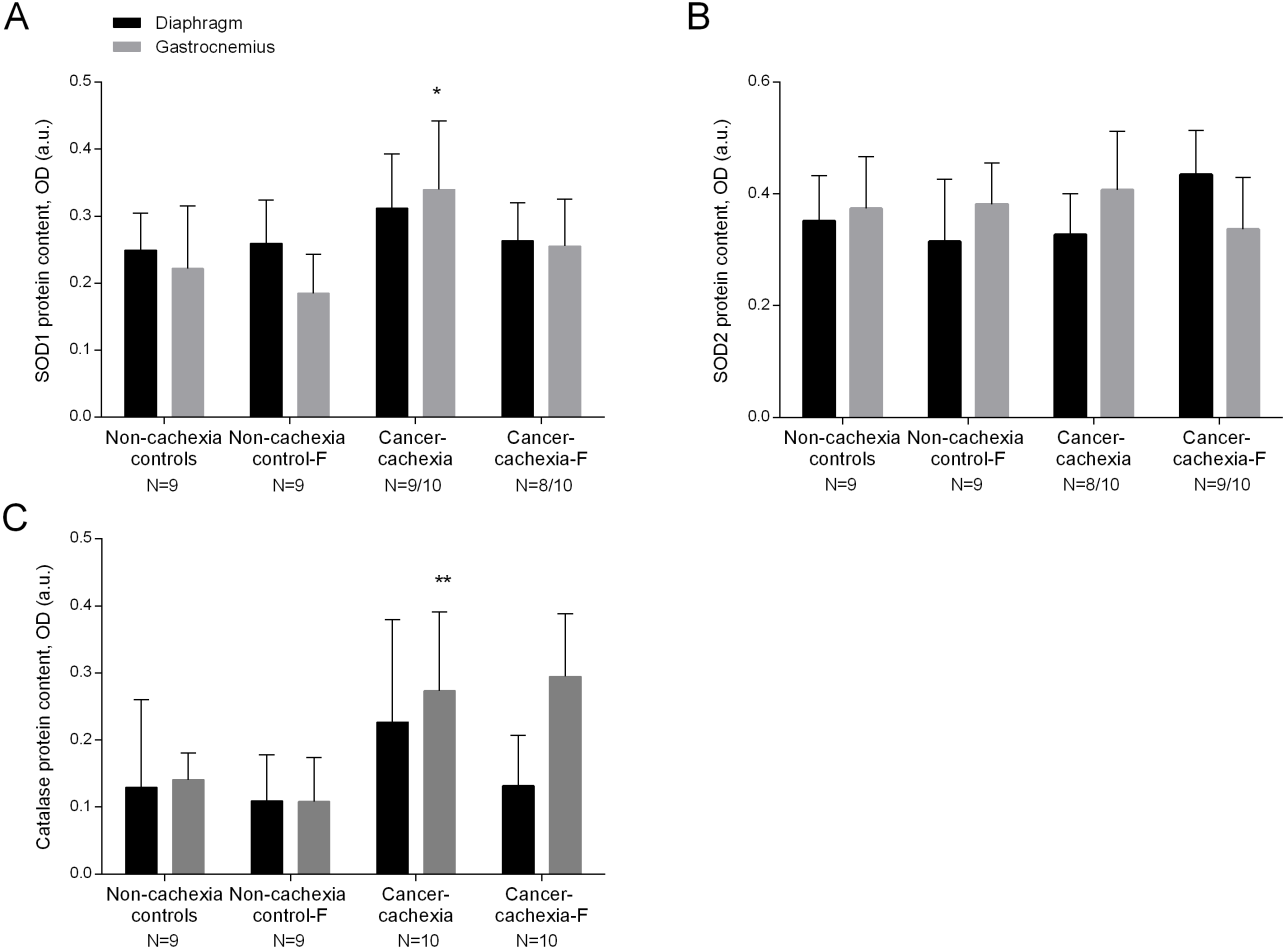
Levels of antioxidants in the diaphragm and gastrocnemius muscles. (A) Mean values and standard deviation of SOD1 protein content in diaphragm (black bars) and gastrocnemius (grey bars) muscles as measured by optical densities in arbitrary units (OD, a.u.). Definition of abbreviations: SOD, superoxide dismutase; F, formoterol. Statistical significance is represented as follow: * *p* ≤ 0.05 between non-cachexia controls and cancer-cachexia rats. (B) Mean values and standard deviation of SOD2 protein content in diaphragm (black bars) and gastrocnemius (grey bars) muscles as measured by optical densities in arbitrary units (OD, a.u.). Definition of abbreviations: SOD, superoxide dismutase; F, formoterol. (C) Mean values and standard deviation of catalase protein content in diaphragm (black bars) and gastrocnemius (grey bars) muscles as measured by optical densities in arbitrary units (OD, a.u.). Definition of abbreviations: F, formoterol. Statistical significance is represented as follow: ** *p* ≤ 0.01 between non-cachexia controls and cancer-cachexia rats.

SOD1 levels were significantly higher in the limb muscle of cancer-cachexia rats than those detected in non-cachexia control animals (Fig. 2A and Fig. S6). SOD2 protein levels did not significantly differ between cancer-cachexia and the non-cachexia control rats (Fig. 2B and Fig. S7). Catalase protein content significantly increased in limb muscles of cancer-cachexia rats compared to non-cachexia controls (Fig. 2C and Fig. S8). In cancer-cachexia rats, treatment with formoterol did not significantly modify protein SOD1, SOD2, or catalase levels in either diaphragm or gastrocnemius muscles (Figs. 2A–2C and Figs. S6–S8). No significant differences were seen in any of these markers in respiratory or limb muscles between non-cachexia controls and non-cachexia control-formoterol animals (Figs. 2A–2C and Figs. S6–S8).

### Muscle inflammation

**Figure 3:**
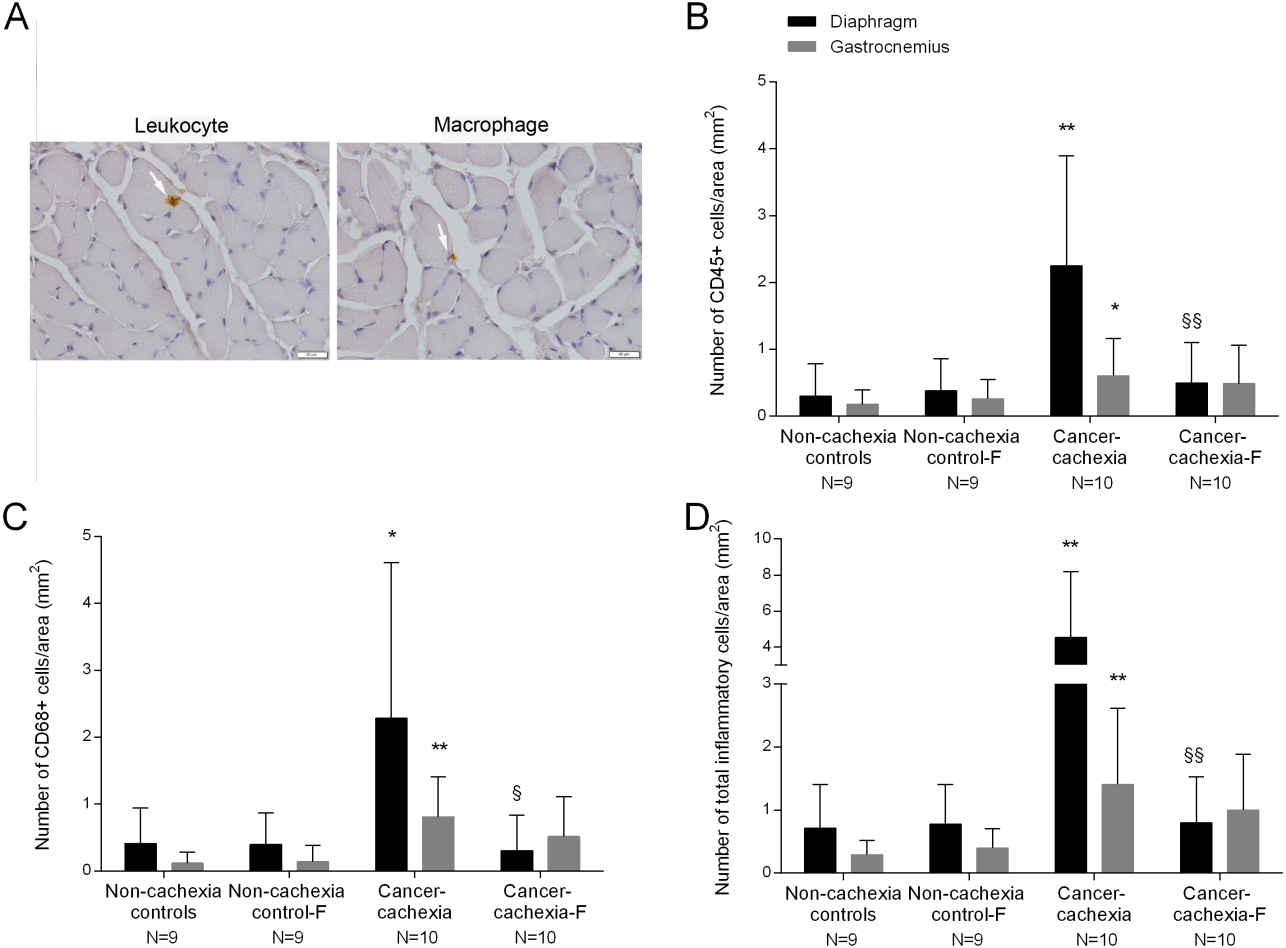
Inflammatory cells in the diaphragm and gastrocnemius muscles. (A) Representative examples of the localitzation of leukocyte and a macrophage with white arrows in the diaphragm of cancer-cachexia group. Note that the concentration of inflammatory cells is extremely low in the muscle cross sections. (B) Mean values and standard deviation of the number CD45+ cells in the diaphragm (black bars) and gastrocnemius (grey bars) muscles as measured by number of CD45+ cells per area (mm^2^). Definition of abbreviations: F, formoterol. Statistical significance is represented as follow: * *p* ≤ 0.05 and ** *p* ≤ 0.01 between non-cachexia controls and cancer-cachexia rats; §§ *p* ≤ 0.01 between cancer-cachexia rats and cancer-cachexia rats treated with formoterol. (C) Mean values and standard deviation of the number CD68+ cells in the diaphragm (black bars) and gastrocnemius (grey bars) muscles as measured by number of CD68+ cells per area (mm^2^). Definition of abbreviations: F, formoterol. Statistical significance is represented as follow: * *p* ≤ 0.05 and ** *p* ≤ 0.01 between non-cachexia controls and cancer-cachexia rats; § *p* ≤ 0.05 between cancer-cachexia rats and cancer-cachexia rats treated with formoterol. (D) Mean values and standard deviation of the number of inflammatory cells (CD45+ and CD68+) in the diaphragm (black bars) and gastrocnemius (grey bars) muscles as measured by number of inflammatory cells per area (mm^2^). Definition of abbreviations: F, formoterol. Statistical significance is represented as follow: ** *p* ≤ 0.01 between non-cachexia controls and cancer-cachexia rats; §§ *p* ≤ 0.01 between cancer-cachexia rats and cancer-cachexia rats treated with formoterol.

The number of infiltrated inflammatory cells (leukocytes and macrophages) was significantly higher in diaphragm and gastrocnemius muscles of cancer-cachexia rats than in non-cachexia controls (Figs. 3A–3D). Treatment with formoterol of cachectic rats attenuated inflammatory cell counts in the study muscles, especially in the diaphragm (Figs. 3A–3D). No significant differences were seen in any of these markers in respiratory or limb muscles between non-cachexia controls and non-cachexia control-formoterol animals (Figs. 3A–3D).

### Specific muscle proteins

**Figure 4:**
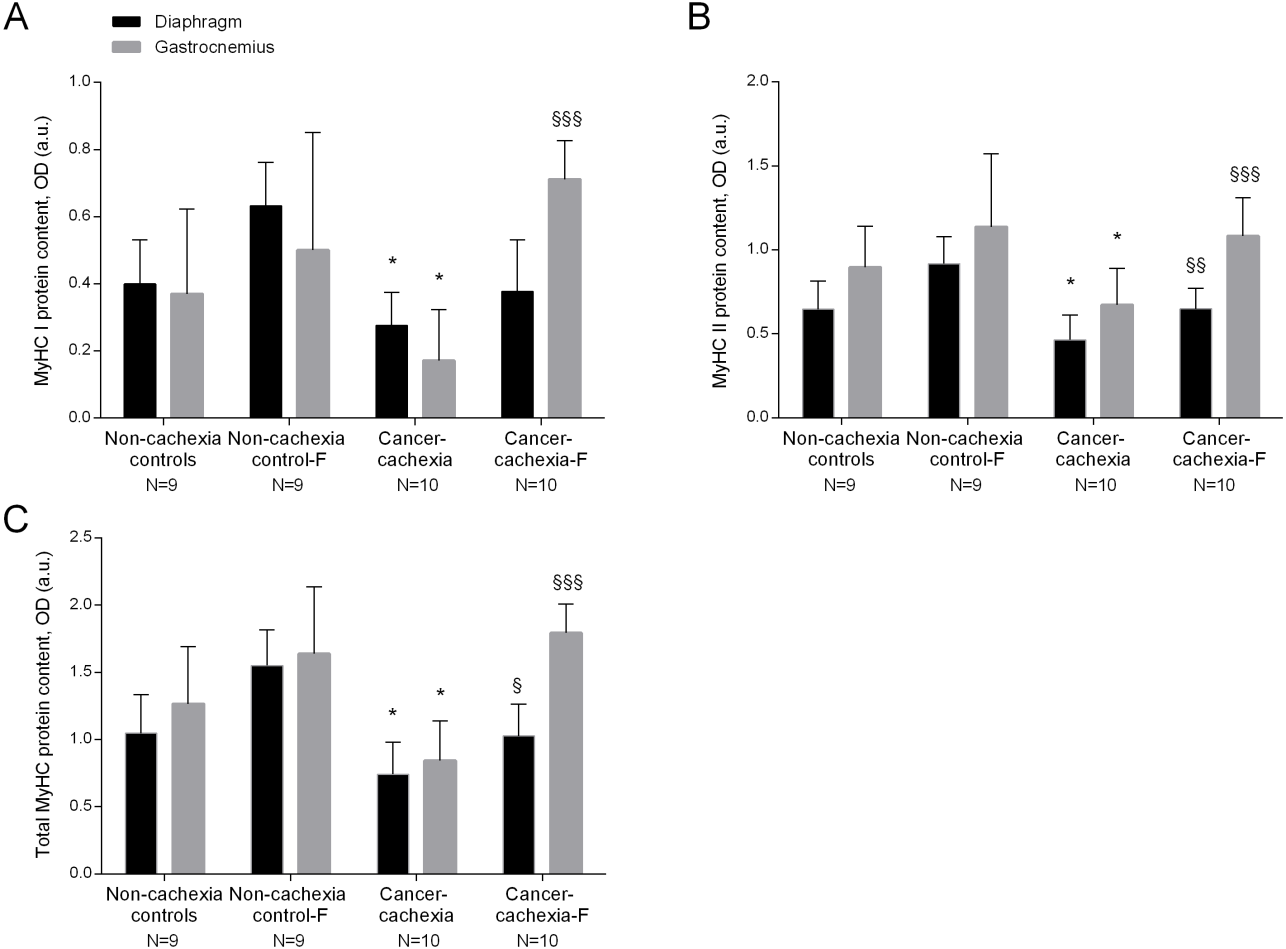
(A) Mean values and standard deviation of MyHC-I protein content in diaphragm (black bars) and gastrocnemius (grey bars) muscles as measured by optical densities in arbitrary units (OD, a.u.). Definition of abbreviations: MyHC, myosin heavy chain; F, formoterol. Statistical significance is represented as follow: * *p* ≤ 0.05 between non-cachexia controls and cancer-cachexia rats; §§§ *p* ≤ 0.001 between cancer-cachexia rats and cancer-cachexia rats treated with formoterol. (B) Mean values and standard deviation of MyHC-II protein content in diaphragm (black bars) and gastrocnemius (grey bars) muscles as measured by optical densities in arbitrary units (OD, a.u.). Definition of abbreviations: MyHC, myosin heavy chain; F, formoterol. Statistical significance is represented as follow: * *p* ≤ 0.05 between non-cachexia controls and cancer-cachexia rats; §§ *p* ≤ 0.01 and §§§ *p* ≤ 0.001 between cancer-cachexia rats and cancer-cachexia rats treated with formoterol. (C) Mean values and standard deviation of total MyHC protein content in diaphragm (black bars) and gastrocnemius (grey bars) muscles as measured by optical densities in arbitrary units (OD, a.u.). Total MyHC protein content was de sum of MyHC-I and MyHC-II protein content. Definition of abbreviations: MyHC, myosin heavy chain; F, formoterol. Statistical significance is represented as follow: * *p* ≤ 0.05 between non-cachexia controls and cancer-cachexia rats; § *p* ≤ 0.05 and §§§ *p* ≤ 0.001 between cancer-cachexia rats and cancer-cachexia rats treated with formoterol.

**Figure 5:**
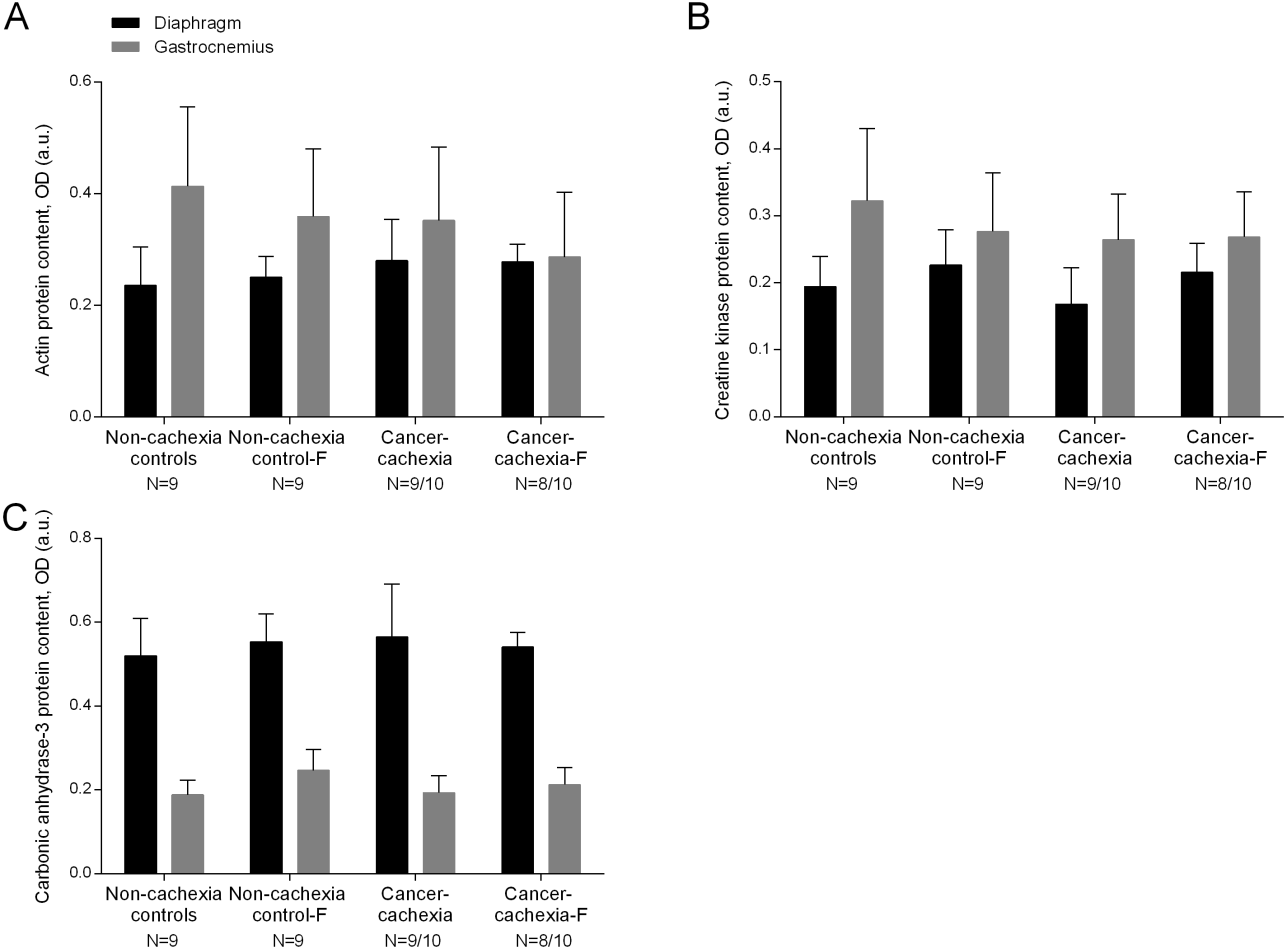
Levels of muscle proteins in the diaphragm and gastrocnemius muscles. (A) Mean values and standard deviation of actin protein content in diaphragm (black bars) and gastrocnemius (grey bars) muscles as measured by optical densities in arbitrary units (OD, a.u.). Definition of abbreviations: F, formoterol. (B) Mean values and standard deviation of creatine kinase protein content in diaphragm (black bars) and gastrocnemius (grey bars) muscles as measured by optical densities in arbitrary units (OD, a.u.). Definition of abbreviations: F, formoterol. (C) Mean values and standard deviation of carbonic anhydrase-3 protein content in diaphragm (black bars) and gastrocnemius (grey bars) muscles as measured by optical densities in arbitrary units (OD, a.u.). Definition of abbreviations: F, formoterol.

In the diaphragm and gastrocnemius muscles of cancer-cachexia rats, protein content of MyHC-I and MyHC-II isoforms, and total MyHC were significantly lower than in muscles of the non-cachexia controls, whereas actin, creatine kinase and carbonic anhydrase-3 protein levels did not significantly differ among the experimental groups (Figs. 4A–4C, 5A–5C and Figs. S9–S13, respectively). Treatment of cancer-cachexia rats with formoterol significantly attenuated the decrease in MyHC-I and MyHC-II isoforms, and total MyHC protein content, while it did not modify the content of the other study muscle proteins (Figs. 4A–4C, 5A–5C and Figs. S9–S13, respectively). No significant differences were seen in any of these markers in respiratory or limb muscles between non-cachexia controls and non-cachexia control-formoterol animals (Figs. 4A–4C, 5A–5C and Figs. S9–S13, respectively).

### MRC complexes: enzyme activities

**Figure 6:**
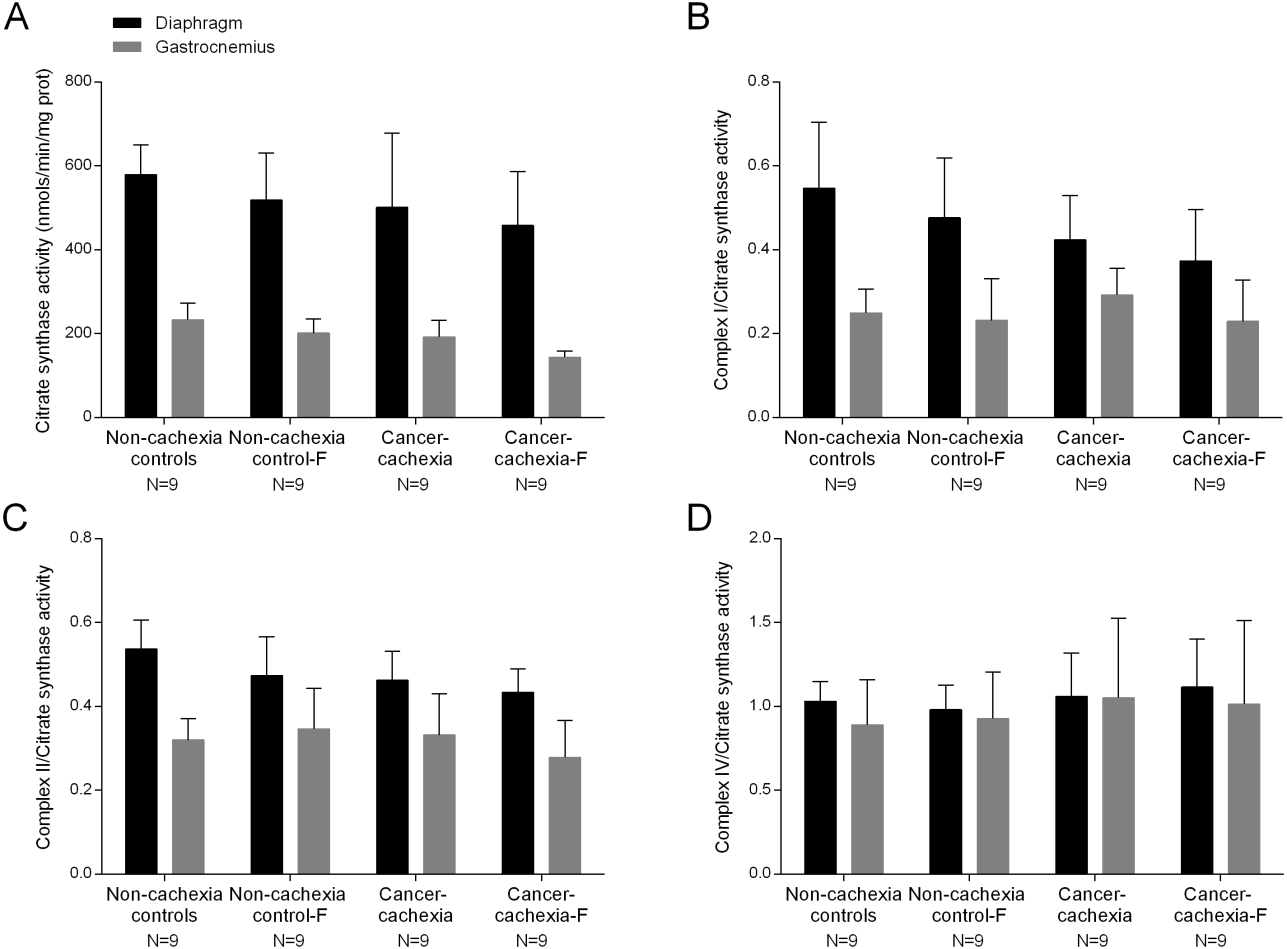
Levels of mitochondrial enzymes in the diaphragm and gastrocnemius muscles. (A) Mean values and standard deviation of citrate synthase activity in the diaphragm (black bars) and gastrocnemius (grey bars) muscles as measured by nanomols per minute per milligram of protein (nmolt/min/mg prot). Definition of abbreviations: F, formoterol. (B) Mean values and standard deviation of the ratio of complex I to citrate synthase activity in the diaphragm (black bars) and gastrocnemius (grey bars) muscles. Definition of abbreviations: F, formoterol. (C) Mean values and standard deviation of the ratio of complex II to citrate synthase activity in the diaphragm (black bars) and gastrocnemius (grey bars) muscles. Definition of abbreviations: F, formoterol. (D) Mean values and standard deviation of the ratio of complex IV to citrate synthase activity in the diaphragm (black bars) and gastrocnemius (grey bars) muscles. Definition of abbreviations: F, formoterol.

In cancer-cachexia rats compared to non-cachexia controls, activity of citrate synthase (CS) did not significantly differ between cancer-cachexia rats and the controls animals in any study muscle (Fig. 6A). Activities of complex I, II and IV did not significantly differ in any of the study muscles among the different experimental groups (Figs. 6B–6D). Treatment with formoterol did not modify activity levels of CS or complex I, II or IV in either diaphragm or gastrocnemius muscles among the study groups (Figs. 6A–6D). No significant differences were seen in any of these markers in respiratory or limb muscles between non-cachexia controls and non-cachexia control-formoterol animals (Figs. 6A–6D).

## Discussion

In the present investigation, the initial hypothesis has been confirmed. Treatment of the cancer cachectic rats with formoterol for seven days attenuated the rise in oxidative stress markers and the decrease in myosin content observed in the study muscles. Another interesting finding was the lack of increased protein oxidation detected in the respiratory muscle of the cancer-cachexia rats. Moreover, alterations in the expression profile of inflammatory cell counts were also attenuated in the muscles of cachectic rodents that were concomitantly treated with formoterol. In view of previously reported findings, the most relevant results encountered in the investigation are discussed below.

### Redox balance markers in respiratory and limb muscles

As previously demonstrated (Busquets et al., 2004; Busquets et al., 2011; Busquets et al., 2012; Marin-Corral et al., 2010; Toledo et al., 2011; Toledo et al., 2016), the Yoshida AH-130 ascites hepatoma is a suitable approach to study the underlying biology of muscle mass loss in cancer cachexia. The tumor growth induces a progressive loss of body weight and skeletal muscle proteins in the host. Our group has published extensively on the elucidation of the biological mechanisms involved in muscle wasting on the basis of this experimental model of oncologic cachexia (Busquets et al., 2004; Busquets et al., 2011; Busquets et al., 2012; Chacon-Cabrera et al., 2014; Chacon-Cabrera et al., 2015; Chacon-Cabrera et al., 2017; Fermoselle et al., 2013; Fontes-Oliveira et al., 2013; Fontes-Oliveira et al., 2014).

The present investigation provides evidence on the posttranslational modifications induced by ROS on proteins in both respiratory and limb muscles and the response to treatment with the beta_2_ agonist formoterol in rats bearing the Yoshida ascites hepatoma. As previously demonstrated in several models of cancer-induced cachexia (Fermoselle, Sanchez & Barreiro, 2011; Marin-Corral et al., 2010; Salazar-Degracia et al., 2016), increased levels of protein oxidation and nitration have also been shown in the gastrocnemius of rats bearing the Yoshida ascites hepatoma in the current study. Importantly, the beta_2_ agonist formoterol attenuated the rise in protein oxidation and nitration levels detected in the limb muscle of the cancer cachectic rats. In fact, increased levels of cAMP were shown to inhibit ROS production and ROS-mediated effects probably via protein kinase A and mitogen-activated protein kinases (MAPK) pathways in tissues such as the vasculature of spontaneously hypertensive rats (Gusan & Anand-Srivastava, 2013).

The enzyme isoforms of SOD catalyze the conversion of two superoxide anions into hydrogen peroxide and molecular oxygen. In the current study, levels of cytosolic SOD1, but not those of SOD2, were significantly greater in the limb muscle of the cancer-cachexia rats than in control animals. These findings are somehow consistent with those previously reported, in which SOD2 levels were not modified in the limb muscles of tumor-bearing rats (Marin-Corral et al., 2010). Nonetheless, they are counter to what shown in mice with lung cancer-induced cachexia in which protein levels of SOD2 were significantly decreased in their respiratory and limb muscles, while levels of SOD1 were not modified by cachexia in the same animals (Chacon-Cabrera et al., 2017). Catalase is a ubiquitous heme protein that is responsible for the detoxification of H_2_O_2_ in tissues along with other enzymes such as glutathione peroxidases and peroxiredoxins. Importantly, the gastrocnemius muscle of the cachectic rats exhibited a significant rise in protein catalase content compared to control animals. A previous investigation also demonstrated a rise in protein catalase content in the fast-twitch fiber muscles of tumor-bearing rats (Marin-Corral et al., 2010). Collectively, these findings suggest that increased protein levels of SOD and catalase were induced probably to offset the deleterious effects of ROS in limb muscles of the cancer-cachexia rats. Interestingly, treatment of the cachectic rats with formoterol attenuated the rise in antioxidants seen in the tumor-bearing rats. It is likely that as cAMP inhibits ROS production (Ryall & Lynch, 2008), upregulation of antioxidants was not required in this model of cancer cachexia.

Levels of reactive carbonyls, MDA- and HNE-protein adducts, and those of protein tyrosine nitration were not modified in the diaphragm of cancer cachectic rats. These findings suggest that lower levels of oxidants were probably produced in the main respiratory muscle compared to the limb muscle in the cancer cachectic rats. Respiratory muscles need to overcome elastic and resistive loads and they are exposed to voluntary and involuntary control. Moreover, the diaphragm needs to contract rhythmically to generate the required forces for ventilation throughout the whole existence of the individuals, whereas the limb muscles are only subject to involuntary control and are not essential to life. The fibers in the diaphragm are unique in that this muscle must continually contract in animals and humans (Zuo et al., 2014; Zuo et al., 2015). On this basis, the differences seen in oxidative stress profile between the gastrocnemius and the diaphragm may be the result of differences in function and activity between these two muscles (Zuo et al., 2014; Zuo et al., 2015). As most of the investigations conducted so far have focused on the study of either respiratory or limb muscles, the current results are of interest. Moreover, in a model of lung carcinogenesis in which cachexia was also induced in mice, oxidative stress markers were also only increased in the gastrocnemius muscle but not in the diaphragm (Salazar-Degracia et al., 2016).

Moreover, species and experimental models may also contribute to the lack of a rise in oxidative stress levels observed in the respiratory muscle of the cachectic rats, since increased protein oxidation has been reported in the diaphragm of lung cancer tumor-bearing mice (Chacon-Cabrera et al., 2017). Additionally, levels of SOD2 were also shown to be reduced in the diaphragm of mice with lung cancer-induced cachexia that were studied for one month (Chacon-Cabrera et al., 2017). A differential metabolic rate of muscle in each model may account for divergences in the expression of oxidative stress markers and antioxidants between types of rodents (mice and rats). Furthermore, the duration and severity of the muscle wasting process in each type of cancer-cachexia model may also partly explain the differences observed in the expression of redox balance markers in the target muscles (Chacon-Cabrera et al., 2017; Fermoselle, Sanchez & Barreiro, 2011; Marin-Corral et al., 2010).

### Proteins susceptible to be modified by oxidative stress and intramuscular inflammation cells

In the study, the content of proteins that have been shown to be carbonylated and affected by muscle mass loss was detected in both respiratory and limb muscles of all groups of mice. Importantly, the content of MyHC (slow- and fast-twitch isoforms) was significantly lower in both respiratory and limb muscles of the cancer cachectic rats and treatment with formoterol attenuated such a decrease. These findings are very consistent with previous reports in which protein levels of contractile myosin were also shown to be significantly reduced in the respiratory (Marin-Corral et al., 2009; Ottenheijm et al., 2005; Ottenheijm et al., 2006) and limb muscles of patients with advanced COPD (Fermoselle et al., 2012; Puig-Vilanova et al., 2014c) and lung cancer cachexia (Puig-Vilanova et al., 2014c) and in both diaphragm and gastrocnemius muscles of cachectic mice with emphysema and/or lung cancer (Chacon-Cabrera et al., 2017; Fermoselle, Sanchez & Barreiro, 2011; Salazar-Degracia et al., 2016).

One of the deleterious effects of oxidative stress in tissues is that involving an increased susceptibility of proteins to be degraded by the proteolytic system. In this regard, we previously demonstrated that levels of carbonylation of MyHC protein were significantly greater in diaphragm (Marin-Corral et al., 2009; Salazar-Degracia et al., 2016) and limb muscles (Fermoselle et al., 2012; Puig-Vilanova et al., 2014c) of patients with advanced COPD than those detected in the controls. As the content of this contractile protein was significantly lower in those muscles, we concluded that oxidative stress may be a trigger for enhanced proteolysis of specific muscle proteins in muscle wasting associated with chronic conditions (Fermoselle et al., 2012; Marin-Corral et al., 2009; Puig-Vilanova et al., 2014c), which could also precede proteolysis in animal models of cancer-induced cachexia (Chacon-Cabrera et al., 2014; Chacon-Cabrera et al., 2017), emphysema (Fermoselle, Sanchez & Barreiro, 2011; Salazar-Degracia et al., 2016), and disuse muscle atrophy (Chacon-Cabrera et al., 2016). Indeed, the proteolytic release of myofilaments is a characteristic feature of muscle wasting conditions (Powers, Kavazis & McClung, 2007).

Levels of contractile actin and the functional proteins creatine kinase and carbonic anhydrase-3 did not significantly differ in any study muscle between cachectic mice and control rats. These findings suggest that despite the previously reported oxidation of contractile actin which may enhance its proteolytic degradation (Powell, Gurzenda & Wahezi, 2001), in our model of cancer-induced cachexia, the content of this protein was not reduced. Levels of creatine kinase, but not carbonic anhydrase-3, were shown to be decreased in diaphragm (Marin-Corral et al., 2009; Ribera et al., 2003) and vastus lateralis (Barreiro et al., 2005; Puig-Vilanova et al., 2014c) of patients with severe COPD and lung cancer cachexia. Differences in the experimental models, humans versus rodents, in the type of conditions, chronic versus rather “acute”, and in the fiber composition of the muscles (Yamashita & Yoshioka, 1991) may account for the lack of effects on creatine kinase protein levels in the cachectic muscles in the current investigation.

In this study, the number of inflammatory cell counts was in general low in muscles all of the groups. Nonetheless, a significant rise in the number of both inflammatory cell types was detected in the diaphragm and gastrocnemius of the cachectic rats and treatment with formoterol attenuated such an increase. These findings are consistent with previous reports in which inflammatory cell counts were also shown to be greater in the respiratory and limb muscles of mice with lung cancer cachexia (Chacon-Cabrera et al., 2014; Chacon-Cabrera et al., 2017). Moreover, in patients with severe COPD, the number of inflammatory cells was also greater in the vastus lateralis muscle than in the healthy controls (Barreiro et al., 2010; Barreiro et al., 2011). The respiratory muscles, however, did not show any significant differences in inflammatory cell counts between patients and the controls (Barreiro et al., 2011). Another interesting observation was that the range of the magnitude of inflammatory cell numbers was higher in the diaphragm than in the limb muscle of the cachectic rats. It is likely that the greater respiratory loads imposed by muscle wasting induced a larger increase in inflammatory cells in the respiratory muscle than the limb muscle of the cachectic rats. In fact, similar trends were also reported in cancer cachectic rodents in previous studies (Chacon-Cabrera et al., 2014; Chacon-Cabrera et al., 2017; Salazar-Degracia et al., 2016).

### MRC activity in respiratory and limb muscles

Interestingly, the magnitude of the measurements of the MRC activity complexes was in general greater in the respiratory than in the limb muscle of the study mice of all groups. These findings were similar to those previously reported in muscles of cancer cachectic mice (Fermoselle et al., 2013). A novelty in the study was the analysis of the activity of complexes I, II, and IV in the diaphragm and gastrocnemius muscles of rats with cancer-induced cachexia. No significant differences were seen between cachectic and control conditions in the study muscles for any of the analyzed complexes, and formoterol did not induce any significant effects. It is likely that the lack of effects on the MRC complexes in the cachectic muscles analyzed in the investigation may account for the absence of modifications resulting from the treatment with the beta_2_ agonist formoterol.

Importantly, these findings are somehow counter to what reported in previous investigations. For instance, in tumor-bearing mice, the activity of MRC complexes I, II, and IV were significantly reduced in both respiratory and limb muscles and treatment with several agents reverted such a decrease to different degrees in each muscle (Fermoselle et al., 2013). Altered expression of proteins involved in mitochondrial biogenesis and fusion were also detected as signs of initiation of cachexia in skeletal muscles through interleukin (IL)-6 regulation in ApcMin/+ mice (White et al., 2012). In the peripheral muscles of cancer cachectic rats, mitochondrial content was also reduced (Fontes-Oliveira et al., 2013; Fontes-Oliveira et al., 2014), while mitochondrial uncoupling was demonstrated in cachectic mice bearing the Lewis lung carcinoma (Tzika et al., 2013). Interestingly, reduced ATP synthesis and mitochondrial disruption events were also detected in limb muscles of cancer-induced cachectic rats (Fontes-Oliveira et al., 2013) and in mice with lung cancer-induced cachexia (Constantinou et al., 2011). Again differences in the metabolic rates of muscles in each experimental model may account for differences observed in mitochondrial structural and biochemical alterations between types of rodents (mice and rats). Furthermore, the duration and severity of the muscle wasting process in each type of cancer-cachexia model may also partly explain the observed differences.

### Study limitations

We acknowledge that the lack of functional data is a limitation in this study. Nonetheless, a first step in this field of investigation was to explore whether oxidative stress levels may be attenuated by treatment with a beta_2_ agonist in both respiratory and limb muscles of rats with cancer cachexia. As shown in former investigations conducted on mice exposed to conditions such as hypoxia and TNF-alpha overexpression (Zuo et al., 2013; Zuo et al., 2014; Zuo et al., 2015), impaired muscle contractility can also be anticipated in the current model. In fact, previous investigations from our group have also revealed that in the same cancer cachexia model, muscle strength and physical activity decreased (Busquets et al., 2011; Toledo et al., 2011), while treatment with formoterol improved those parameters in the rats (Busquets et al., 2011).

## Conclusions

We conclude that in this *in vivo* model of cancer-cachectic rats, the diaphragm is likely to be more protected against oxidant production and oxidative stress than the gastrocnemius muscle. Formoterol treatment of these animals attenuated the rise in oxidative stress in the limb muscles as well as the inflammatory cell infiltration and the loss of myosin protein isoforms seen in both study muscles, whereas no effects were observed in the MRC complex activities. These findings have therapeutic implications as they demonstrate beneficial effects of this beta_2_ agonist through decreased protein oxidation and inflammatory events in cachectic muscles, especially the gastrocnemius.

##  Supplemental Information

10.7717/peerj.4109/supp-1 Supplemental Information 1 Clean version of the revised online data supplement

10.7717/peerj.4109/supp-2 Supplemental Information 2 Database of the last version of the manucript

## Additional Information and Declarations

### Data Availability

The following information was supplied regarding data availability: The raw data has been provided as a Supplemental File.
