# Supplementary material for: Formoterol attenuates increased oxidative stress and myosin protein loss in respiratory and limb muscles of cancer cachectic rats
Source: PeerJ. 2017 Dec 13;5:e4109. doi: 10.7717/peerj.4109 (PMC5732544; doi:10.7717/peerj.4109)
Supplement: Supplemental Information 1 [file peerj-05-4109-s001.docx]

**Online supplementary material**

**FORMOTEROL ATTENUATES increased OXIDATIVE STRESS and myosin protein loss IN RESPIRATORY AND LIMB MUSCLES of cancer cachectic RATS**

**Anna Salazar-Degracia, Sílvia Busquets, Josep Argilés, Francesc López-Soriano, and Esther Barreiro**

**MATERIALS AND METHODS**

**Animal experiments**

*Experimental design.* Male Wistar rats (5 weeks, 130-165 grams, Interfauna, Barcelona, Spain) were used for the purpose of the investigation. Animals were randomly subdivided into four groups (N= 10/group) and were studied for seven days: 1) non-cachexia controlss, 2) non-cachexia controlss treated with formoterol (non-cachexia controls-F), 3) cancer-cachexia rats, and 4) cancer-cachexia rats treated with formoterol (cancer-cachexia-F). Cachexia was induced as a result of an intraperitoneal inoculum of 10^8^ AH-130 Yoshida ascites hepatoma cells, which were obtained from tumors in exponential growth as previously described (Busquets et al, 2004;Busquets et al, 2011;Busquets et al, 2012;Fontes-Oliveira et al, 2013;Fontes-Oliveira et al, 2014;Toledo et al, 2011;Toledo et al, 2016). AH-130 Yoshida ascites hepatoma is a well-validated model characterized by a rapid and progressive loss of body weight and muscle mass. As previously demonstrated (Lopez-Soriano et al, 1997;Toledo et al, 2011), a moderate cachexia (8% of body weight loss) was already seen on day 4, while reaching 20-25% of body weight loss on day 7. For ethical reasons (large tumor sizes), seven days was established as the duration of the study.

Formoterol treatment was administered subcutaneously (0.3 mg/kg body weight/24h, dissolved in physiological solution) six hours after inoculation of the tumor cells and was thereafter administered daily during seven consecutive days up until the sacrifice of the animals. Non-treated rats received the corresponding volume of physiological solution that was administered subcutaneously every day for seven days (Busquets et al, 2004;Busquets et al, 2011;Busquets et al, 2012;Fontes-Oliveira et al, 2013;Fontes-Oliveira et al, 2014;Toledo et al, 2011;Toledo et al, 2016). In this model of cancer cachexia, the most suited dose and duration of the formoterol treatment was established in previous investigations of our group (Busquets et al, 2004;Toledo et al, 2014). Importantly, administration of 0.3 mg/kg/24h formoterol for seven days was the minimum dose that ensured a beneficial anti-cachectic effect in the rats, while no side-effects were induced on other organs including the heart (Busquets et al, 2004;Toledo et al, 2014).

All animal experiments were conducted at *Facultat de Biologia, Universitat de Barcelona (Barcelona)*. This was a controlled study designed in accordance with both the ethical standards on animal experimentation in our institution (EU 2010/63 CEE and *Real Decreto* 53/2013 BOE 34, Spain) and the Helsinki convention for the use and care of animals. Ethical approval was obtained by the institutional Animal Experimentation Ethics Committee (reference number DAAM: 8315, *Universitat de Barcelona*).

**In vivo measurements in the animals**

Food and water were administered *ad libitum* to the animals for the entire duration of the study. All the animals were maintained at a temperature of 22 ± 2 °C with a regular light-dark cycle (lights were on from 08:00 a.m. to 08:00 p.m.) and had free access to food and water. Body weight was determined in all animals on day 0 and prior to their sacrifice on day 7. Tumor weights were determined in all animals upon sacrifice. The percentage of body weight gain at the end of the period was calculated as follows: [(body weight on day 7 – tumor weight on day 7) – body weight on day 0]/ body weight on day 0 x 100 (Busquets et al, 2004;Busquets et al, 2011;Busquets et al, 2012;Fontes-Oliveira et al, 2013;Fontes-Oliveira et al, 2014;Toledo et al, 2011;Toledo et al, 2016).

**Sacrifice and sample collection**

On day 7 after tumor transplantation, the animals were weighed and anesthetized through an intraperitoneal injection of 3:1 ketamine/xylazine mixture (Imalgene^®^ 1000, Rhone Merieux, France and Rompun^®^, Bayer AG, Leverkusen, Germany, respectively). In all animals, the pedal and blink reflexes were evaluated in order to verify total anesthesia depth. The diaphragm and gastrocnemius muscles were obtained from all the animals. In all samples, muscle specimens were immediately frozen in liquid nitrogen and subsequently stored at -80ºC. Frozen tissues were used to assess the expression of the target molecular markers (Busquets et al, 2004;Busquets et al, 2011;Busquets et al, 2012;Fontes-Oliveira et al, 2013;Fontes-Oliveira et al, 2014;Toledo et al, 2011;Toledo et al, 2016).

**Muscle biology analyses**

All the muscle biological experiments were performed in the laboratory at *Hospital del Mar-*IMIM*-Universitat Pompeu Fabra* (Barcelona).

*Detection of reactive carbonyls in muscle proteins*. Changes in protein carbonylation in crude muscle homogenates were detected using the commercially available Oxyblot kit (Chemicon International Inc., Temecula, CA, USA). Carbonyl groups in the protein side chains were derivatized to 2,4-dinitrophenylhydrazone (DNP) by reaction with 2,4-dinitrophenylhydrazine (DNPH) according to the manufacturer’s instructions and previous methodologies (Barreiro et al, 2012). Briefly, 15 micrograms of protein were used per derivatization reaction. Proteins were then denatured by addition of 12% sodium dodecylsulfate (SDS). The samples were subsequently derivatized by adding 10 microL DNPH solution and incubated for 20 minutes. Finally, 7.5 microL neutralization solution and 2-mercaptoethanol were added to the sample mixture. DNP-derivatized proteins were loaded onto 12% Tris-glycine sodium dodecylsulfate polyacrylamide gels (SDS-PAGE) and separated by electrophoresis.

*Immunoblotting.* The effects of ROS and RNS on muscle proteins were explored using previously published methodologies (Chacon-Cabrera et al, 2014;Chacon-Cabrera et al, 2015;Chacon-Cabrera et al, 2016b;Chacon-Cabrera et al, 2016a;Fermoselle et al, 2012;Puig-Vilanova et al, 2014;Salazar-Degracia et al, 2016). Briefly, frozen muscle specimens from the diaphragm and gastrocnemius muscles of all the experimental groups were homogenized in a specific buffer containing 50 mM 4-(2-hydroxyethyl)-1-piperazineethanesulfonic acid (HEPES), 150 mM NaCl, 100 mM NaF, 10 mM Na pyrophosphate, 5 mM ethylenediaminetetraacetic acid (EDTA), 0.5% Triton-X, 2 micrograms/mL leupeptin, 100 micrograms/mL phenylmethylsulfonyl fluoride (PMSF), 2 micrograms/mL aprotinin, and 10 micrograms/mL pepstatin A. Samples were then centrifuged at 1,000 g for 30 minutes. MyHC-I and MyHC-II isoforms were detected in the immunoblots from muscle homogenates, in which the myofibrilar compartment was obtained using a specific buffer containing 0.5 M NaCl, 20 mM Na pyrophosphate, 50nM Tris, 1mM EDTA, and 1mM dithiothreitol (DTT). The homogenates were obtained by centrifugation of the samples at 2,500 g for 10 minutes (Picard et al, 2011). In the muscle homogenates, the pellet was discarded and the supernatant was designated as a crude cytoplasmic homogenate. The entire procedures were always conducted at 4ºC. Protein levels in crude muscle homogenates were spectophotometrically determined using the Bradford method in triplicates for all the samples and bovine serum albumin (BSA) was used as the standard (Bio-Rad protein reagent, Bio-Rad Inc., Hercules, CA, USA). The final protein concentration in each sample was calculated from at least two Bradford (Bradford, 1976) measurements that were almost identical. Equal amounts of total protein (ranging from 5 to 30 micrograms, depending on the antigen and antibody) from crude muscle homogenates were always loaded onto the gels, as well as identical sample volumes/lane. For the sake of comparisons among the different experimental groups, muscle sample specimens were always run together and kept in the same order. Oxidative stress and antioxidant markers were analyzed independently, in two different sets of samples for diaphragm and gastrocnemius muscles from all groups of rats. Experiments were confirmed at least twice for all the antigens quantified in the study. Fresh gels were specifically loaded for each of the antigens in all cases.

Proteins were then separated by electrophoresis, transferred to polyvinylidene difluoride (PVDF) membranes, blocked with 5% non-fat milk or with 1% BSA, and incubated with the corresponding primary antibodies overnight for each of the target markers. Protein content of the different markers was identified using specific primary antibodies: anti-DNP moiety antibody (rabbit anti-DNP antibody from the Oxyblot kit), 4-hydroxy-2-nonenal (HNE)-protein adducts (anti-HNE-protein adducts antibody, Alpha Diagnostic International, San Antonio, TX, USA), malondialdehyde (MDA)-protein adducts (anti-MDA-protein adducts antibody Academy Bio-Medical Company, Inc., Houston, TX, USA), 3-nitrotyrosine (anti-3-nitrotyrosine antibody, invitrogen, Eugene, Oregon, USA), superoxide dismutase (SOD)2 (anti-SOD2 antibody, Santa Cruz), SOD1 (anti-SOD1 antibody, Santa Cruz), catalase (anti-catalase antibody, Calbiochem, Darmstadt, Germany), MyHC-I (anti-MyHC-I antibody, Sigma-Aldrich, St. Louis, MO, USA), MyHC-II (anti-MyHC-II antibody, Abcam, Cambridge, UK), actin (anti-alpha-sarcomeric actin antibody, clone 5C5, Sigma Sigma-Aldrich, St. Louis, MO, USA), creatine kinase (anti-creatine kinase antibody, Santa Cruz), carbonic anhydrase-3 (anti-carbonic anhydrase-3 antibody, Santa Cruz Biotechnology, Santa Cruz, CA, USA), and glyceraldehyde-3-phosphate dehydrogenase (GAPDH, anti-GAPDH antibody, Santa Cruz).

Specific proteins from all samples were detected using horseradish peroxidase (HRP)– conjugated secondary antibodies (Jackson ImmunoResearch Inc, West Grove, PA, USA) and a chemiluminescence kit (Thermo scientific, Rockford, IL, USA). The specificity of anti-MyHC-I and anti-MyHC-II antibodies was tested by running independent immunoblots containing slow- and fast-twitch muscles from both rats and humans as follows: diaphragm, gastrocnemius, extensor digitorium longus and soleus from rats and diaphragm and vastus lateralis from humans (data not shown). The specificity of all the other antibodies was confirmed by omission of the primary antibody and incubation of the membranes only with secondary antibodies. PVDF membranes were scanned using the Molecular Imager Chemidoc XRS System (Bio-Rad Laboratories, Hercules, CA, USA) and the software Quantity One version 4.6.5 (Bio-Rad Laboratories). For each of the study antigens, PVDF membranes of samples from the different groups were always detected in the same picture under identical exposure times. Optical densities of specific proteins were quantified using the software Image Lab version 2.0.1 (Bio-Rad Laboratories).

Values of total reactive carbonyl groups, total HNE- and MDA-protein adducts, and protein tyrosine nitration in a given sample were calculated by addition of the optical densities (arbitrary units) of individual protein bands in each case. Final optical densities obtained in each specific group of animals corresponded to the mean values of the different samples (lanes) of each of the study antigens. Values of total MyHC were the sum of the optical densities obtained from the MyHC-I and MyHC-II immunoblots separately. To validate equal protein loading across lanes, the glycolytic enzyme GAPDH was used as the protein loading control in all the immunoblots, except for MyHC-I and MyHC-II isoforms, in which Coomassie Blue staining was used, since the long electrophoresis required to detect these antigens did not enable the identification of small-size proteins such as GAPDH (Figures S1-S4 and S6-S13, respectively). Standard stripping methodologies were employed to detect the protein loading control GAPDH for each of the markers, as well as to detect MyHC-II protein band in the membranes that had been previously incubated with the anti-MyHC-I antibody. Briefly, membranes were stripped of primary and secondary antibodies through incubation with a stripping solution (25 nM glycine, 1% SDS, pH 2.0) for 30 minutes followed by two consecutive washes containing phosphate buffered saline with tween (PBST) at room temperature for 10 minutes. Subsequently, membranes were blocked with either 5% non-fat milk or 1% BSA, depending on the primary antibody and reincubated with primary and secondary antibodies following the procedures described above.

*Identification of carbonylated muscle proteins using 2D electrophoresis.* Following procedures previously published (Marin-Corral et al, 2009;Marin-Corral et al, 2010), two-dimensional gel electrophoresis was used to separate and identify the carbonylated proteins in the gastrocnemius muscles of all groups of rats. These experiments were carried out in the limb muscle as protein carbonylation levels were only significantly increased in the gastrocnemius of the cancer cachectic rats. Briefly, 4 volumes of 10 mM DNPH were first added (reactive carbonylated proteins) to crude muscle homogenates (400 micrograms protein/sample) and incubated for 30 minutes at room temperature in order to specifically identify carbonylated proteins. The reaction was stopped by adding the neutralization solution. In both cases, crude muscle homogenates (400 micrograms protein/sample) were prepared for 2D-electrophoresis with the 2D Clean-up kit (Amersham Biosciences, Piscataway, NJ, USA) following the manufacturer’s instructions. The samples were then incubated for 15 minutes on ice, centrifuged for 5 minutes at 13,000 g and the pellets were then washed three times and centrifuged at 13,000 g for 5 minutes. The pellets were re-suspended in 2D re-hydration buffer (8 M urea, 2% 3-[(3-cholamidopropyl)dimethylammonio]-1-propanesulfonate (CHAPS), 20 mM DTT, and 0.002% bromophenol blue). Each muscle sample was then separated into two portions (200 micrograms total each) and both portions underwent 2-D electrophoresis.

First-dimensional protein separation was performed with the the Ettan IPGPhor 3 (GE Healthcare Biosciences AB, Uppsala, Sweden). Samples were applied to immobilized pH gradient (IPG) strips (18-cm nonlinear pH 3-10, GE Healthcare Biosciences AB, Uppsala, Sweden) for 30 minutes at room temperature. The strips were then covered with mineral oil overnight and isoelectric focusing was performed at a maximum of 10,000 V/ hour for up to a total of 35,200 V-hour. For the second dimension, the IPG strips were equilibrated at room temperature for 30 minutes in equilibration buffer (6 M urea, 2% SDS, 50 mM Tris-HCl, 30% glycerol, and 0.002% bromophenol blue) to which 1% DTT was added prior to use. An additional 30 minutes equilibration period was then used with equilibration buffer to which 2.5% iodoacetamide was added. The strips were then embedded in 0.5% agarose on the top of 30% acrylamide gels. The second dimension SDS/PAGE was performed for 5 hours, 70 mA per gel at 250 V. One of the resulting 2D gels for each muscle sample was then stained with silver stain. Gels were fixed for an hour in a fixation solution (50% acetic acid, 50% methanol), then rinsed twice in water, sensitized for one minute in 0.2% sodium thiosulfate, followed by rinsing in water and immersion for 30 minutes in a silver nitrate solution (2% silver nitrate). Gels were then rinsed twice in water and developed in a developer solution (20% sodium carbonate, 0.05% formaldehyde, 0.004% sodium thiosulfate). A stop solution (6% acetic acid) was then added for 15 minutes followed by rinsing with water for 5 minutes. Gels were then stored in 1% acetic acid. The second gel derived from a given sample underwent electrophoretical transfer to PVDF membrane and immunoblotting with anti-DNP antibody as described above. Gels and PVDF membranes were imaged with a digital camera and aligned (Adobe Photoshop 8.0.1, San Jose, CA, USA) so as to identify positive spots of either carbonylated proteins on the gels. Optical densities (arbitrary units) of the immunoblot spots corresponding to each oxidatively modified protein were measured in each muscle of all groups of animals using the software Quantity One analysis version 4.6.5 (Bio-Rad Laboratories, Philadelphia, PA, USA) (Busquets et al, 2004;Busquets et al, 2011;Busquets et al, 2012;Fontes-Oliveira et al, 2013;Fontes-Oliveira et al, 2014;Toledo et al, 2011;Toledo et al, 2016).

*Identification of carbonylated proteins using mass spectrometry (MS).* Identification of carbonylated proteins using mass spectrometry was conducted in the Proteomics Laboratory at *Universitat Pompeu Fabra (Barcelona)* following the quality criteria established by *ProteoRed* standards (*Instituto Nacional de Proteómica, Spain*) and procedures previously published (Marin-Corral et al, 2009). Protein carbonylated spots from silver-stained gels were manually excised for in-gel digestion in a 96-well ZipPlate placed in a Multiscreen vacuum manifold (Millipore, Billerica, ME, USA). Proteins were reduced, alkylated, and digested with sequence grade trypsin (Promega, Madison, WI, USA). Peptides were eluted with 15-25 microL of 0.1% trifluoroacetic acid (TFA) in 50% acetonitrile (ACN). 2.5 microL of tryptic digest were deposited onto Mass·Spec·Turbo 192 type 1 peptide chips pre-spoted with alpha-cyano-4-hydroxycinnamic acid (CHCA) (Qiagen, Germantown, MD, USA) and left for 3 minutes for peptide adsorption. Then each spot was washed for 5 seconds with one microL of finishing solution (Qiagen, Germantown, MD, USA) and left until dryness. Matrix assisted laser desorption/ionization time-of-flight (MALDI-TOF) MS was performed in a Voyager DE-STR instrument (Applied Biosystems, Foster City, CA, USA) using a 337-nm nitrogen laser and operating in the reflector mode, with an accelerating voltage of 20 kV. Samples were analyzed in the m/z 800-3000 range and were calibrated externally using a standard peptide mixture (Sequazyme Peptide Mass Standards kit, Applied Biosystems, Foster City, CA, USA). Peptides from trypsin autolysis were used for the internal calibration. Protein identification from MALDI-TOF results was done with the MASCOT search engine (Matrix Science, London, UK) using human proteins available in the SwissProt database as well in the NCBI non-redundant database for the identification of the *Rattus novergicus* proteins. Moreover, the following parameters were used for database searches: one missed cleavage allowed, plus Cys carbamidomethylation as fixed and Met oxidation as variable modifications selected, respectively (Marin-Corral et al, 2009).

*Intramuscular cellular inflammation.* In order to evaluate the presence of inflammatory cells in diaphragm and gastrocnemius muscles, immunohistochemical analyses were conducted in each muscle of all study groups following previously published methodologies (Barreiro et al, 2011). On three-micrometer muscle paraffin-embedded sections, leukocytes (anti-CD45 antibody, clone 2B11 & PD7/26, Dako Cytomation Inc., Carpinteria, CA) and macrophages (anti-CD68 antibody, clone PG-M1, Dako Cytomation Inc.) were identified following immunohistochemical procedures. After incubation with the corresponding primary antibodies, slides were washed and incubated for 30 minutes with biotinylated universal secondary antibody followed by incubation (30 minutes) with HRP-conjugated streptavidin and diaminobenzidine (kit LSAB + HRP. Dako Cytomation Inc.) as a substrate. Slides were counterstained with hematoxylin, dehydrated and mounted for conventional microscopy. Total number of both leukocytes and macrophages were counted in all muscle preparations of all animal groups under light microscopy (Olympus BX 61, Olympus Corporation, Tokyo, Japan) coupled with an image-digitizing camera (Olympus DP 71, Olympus Corporation). Furthermore, the total area in each muscle section was also determined using the light microscope, the image-digitizing camera, and Image J software (National Institute of Health, available at <http://rsb.info.nih.gov/ij/>). Results corresponding to inflammatory cell counts were expressed as follows: the ratio of either leukocyte or macrophage numbers to total muscle section area and the ratio of both cell types to total muscle section area in both diaphragm and gastrocnemius muscles (Barreiro et al, 2011).

**Mitochondrial respiratory chain (MRC) complexes: enzyme activities in muscles**

*Homogenization procedures.* Snap-frozen diaphragm and gastrocnemius muscles obtained from all rats were homogenized using a Homogenisator Potter S (Sartorius Stedim Biotech GmbH, Goettingen, Germany) following previously published methodologies (Fermoselle et al, 2013;Medja et al, 2009). Muscle homogenization was always performed between 0ºC and 6ºC. In general, 20-50 mg of whole muscle tissue were placed into the homogenizer containing 9 volumes (w/v) manitol buffer pH 7.2 (225 mM manitol, 75 mM sucrose, 10 mM Tris HCl, 0.1 mM EDTA), at 94 g, while three strokes up and down were performed. Samples were then transferred onto a new tube and centrifuged at 650 g for 20 minutes. After centrifugation, the supernatants were collected and placed into new tubes, while the pellets were re-suspended with the initial manitol buffer volume, pH 7.2. The homogenization procedure was repeated entirely with the resuspended pellets. The supernatants obtained from the second centrifugation were added to the first one, thus yielding the final sample supernatants. Protein concentrations were measured using the Bradford technique (Bradford, 1976).

*Mitochondrial citrate synthase (CS) activity.* The procedures employed to determine CS activity have also been previously reported (Fermoselle et al, 2013;Medja et al, 2009). CS catalyzes the condensation of acetyl-coenzyme-A with oxaloacetate resulting in citrate and coenzyme A. The latter compound can be measured using 5,5′-Dithiobis (2-nitrobenzoic acid) (DTNB). For each sample, 930 microL of reaction medium (100 microM DTNB, 300 microM acetyl coenzyme A (CoA), 0.1% Triton x 100, 100 mM Tris HCl pH 8.1) and 20 micrograms of final supernatant were added to the spectrophotometer cuvettes. The reaction was incubated at 37ºC inside the spectrophotometer (Ultrospec 2100 pro, Amersham Biosciences, Piscataway, NJ, USA) for 5 minutes. Baseline absorbances were read at 412 nm every 15 seconds for 4 minutes. The reaction was then initiated by the addition of 50 microL 10 mM oxaloacetate in 100 mM Tris HCl pH 8.1 to the cuvettes. The reaction was monitored at 412 nm every 15 seconds for 4 minutes. The Absorbance / minute of the baseline measurements were subtracted from the Absorbance / minute of the reaction measurements. The specific activity of CS enzyme was expressed as nanomoles per minute per mg of protein. Intra- and inter-assay coefficients of variation (CV) were 5.07 and 2.7%, respectively.

*Mitochondrial Complex I Activity.* All procedures employed in the current investigation have been published previously (Fermoselle et al, 2013;Medja et al, 2009). Enzymatic of nicotinamide adenine dinocleotide (NADH) ubiquinone oxidoreductase rotenone sensitive is based on the oxidation rate of NADH in the presence of rotenone. The rotenone-resistant activity was substracted from the total activity of NADH ubiquinone oxidoreductase to obtain the activity sensitive to rotenone. For each sample, 2046 microL of reaction medium (50 mM potassium phosphate (KP) pH 7.5, 100 microM Decylubiquinone, 3.75 mg/mL BSA) and 40 micrograms of postnuclear supernatant were included and mixed in a tube. Two spectrophotometer cuvettes were measured for each sample: in the first one, 950 microL of the mixture were poured, while in the second, 950 microL of the mixture plus 5 microL of 2.5 mM rotenone were poured. Both cuvettes were incubated at 37ºC for 5 minutes inside the spectrophotometer. The reaction was initiated by adding 50 microL of 2 mM NADH to each cuvette. Absorbances were measured at 340 nm every 15 seconds for 3 minutes. The Absorbance / minute of the cuvette with rotenone was subtracted to the Absorbance / minute of the cuvette without rotenone. The specific activity of complex I was expressed as nanomoles per minute per mg of protein. Additionally, the ratio of complex I to CS activities was also evaluated in both diaphragm and gastrocnemius muscle. Intra- and inter-assay coefficients of variation were 8.93 and 7.84%, respectively.

*Mitochondrial Complex II Activity.* All these procedures have been previously reported (Fermoselle et al, 2013;Medja et al, 2009). Succinate decylubiquinone reductase activity was measured using 2,6-dichlorophenolindophenol (DCPIP) as the electron acceptor. Nine hundred and seventy-six microL of reaction medium (25 mM KP pH 7.5, 20 mM succinate, 50 microM DCPIP, 1 mM potassium cyanide (KCN), 100 microM adenosine-5'-triphosphate (ATP), 2 mg/mL BSA) and 20 micrograms of sample postnuclear supernatant were added to the spectrophotometer cuvettes. The reactions were incubated at 37ºC for 5 minutes inside the spectrophotometer. Baseline measurements were first determined as follows: absorbances were measured at 600 nm every 15 seconds for 3 minutes, and the reaction was initiated by adding 4 microL of 25 mM decylubiquinone to the cuvettes. Absorbances were then measured at 600 nm every 15 seconds for 3 minutes. The Absorbance / minute from the baseline measurements were subtracted from the Absorbance / minute obtained after addition of decylubiquinone. The specific activity of complex II was expressed as nanomoles per minute per mg of protein. Moreover, the ratio of complex II to CS activities was also evaluated in both muscles. Intra- and inter-assay coefficients of variation were 7.81 and 5.18%, respectively.

*Mitochondrial Complex IV Activity.* All procedures used for measuring this complex have been published previously (Fermoselle et al, 2013;Medja et al, 2009). Complex IV activity was measured using reduced cytochrome c as the substrate. Fifteen mL of initial solution (100 microM cytochrome c in 50 mM KP pH 7.0 solution) were prepared. 100% oxidized solution was prepared by adding 20 mg potassium ferricyanide into 1 mL of initial solution. 100% reduced solution was prepared by adding 20 mg of sodium hydrosulfite into 3 mL of initial solution. 100% reduced solution absorbance was measured using the 100% oxidized solution as the blank. Reduced initial solution was prepared as indicated: small aliquots of 100% reduced solution were added to the initial solution until its absorbance reached the 90-95% of the 100% reduced solution absorbance. For each sample, 980 microL of reduced initial solution were added to the spectrophotometer cuvettes, and were incubated at 37ºC for 5 minutes inside the spectrophotometer. The reaction was then initiated by adding 20 micrograms of postnuclear supernatant to each cuvette. Absorbances were measured at 550 nm every 15 seconds for 3 minutes. The specific activity of complex IV was expressed as nanomoles per minute per mg of protein. Additionally, the ratio of complex IV to CS activities was also evaluated in both diaphragm and gastrocnemius muscles. Intra- and inter-assay coefficients of variation were 4.13 and 4.03%, respectively.

**Statistical Analysis**

The normality of the study variables was verified using the Shapiro-Wilk test. Results are presented as mean (standard deviation). The comparisons between the different study groups were analyzed using the one-way analysis of variance (ANOVA), in which Tukey’s post hoc analysis was used to adjust for multiple comparisons. The sample size chosen was based on previous studies (Busquets et al, 2004;Busquets et al, 2011;Busquets et al, 2012;Chacon-Cabrera et al, 2014;Chacon-Cabrera et al, 2015;Chacon-Cabrera et al, 2016b;Chacon-Cabrera et al, 2016a;Fermoselle et al, 2011;Fermoselle et al, 2013;Fontes-Oliveira et al, 2013;Fontes-Oliveira et al, 2014;Marin-Corral et al, 2010;Salazar-Degracia et al, 2016;Toledo et al, 2011;Toledo et al, 2016) and on assumptions of 80% power to detect an improvement of more than 20% in measured outcomes at a level of significance of *P*≤ 0.05. All statistical analyses were performed using the Statistical Package for the Social Sciences (Portable SPSS, PASW statistics 18.0 version for windows, SPSS Inc., Chicago, IL, USA).

**Literature Cited**

**Barreiro E, del Puerto-Nevado L, Puig-Vilanova E, Perez-Rial S, Sanchez F, Martinez-Galan L, Rivera S, Gea J, Gonzalez-Mangado N, Peces-Barba G (2012). Cigarette smoke-induced oxidative stress in skeletal muscles of mice. Respir Physiol Neurobiol 182:9-17.**

**Barreiro E, Ferrer D, Sanchez F, Minguella J, Marin-Corral J, Martinez-Llorens J, Lloreta J, Gea J (2011). Inflammatory cells and apoptosis in respiratory and limb muscles of patients with COPD. J Appl Physiol (1985 ) 111:808-817.**

**Bradford MM (1976). A rapid and sensitive method for the quantitation of microgram quantities of protein utilizing the principle of protein-dye binding. Anal Biochem 72:248-254.**

**Busquets S, Figueras MT, Fuster G, Almendro V, Moore-Carrasco R, Ametller E, Argiles JM, Lopez-Soriano FJ (2004). Anticachectic effects of formoterol: a drug for potential treatment of muscle wasting. Cancer Res 64:6725-6731.**

**Busquets S, Toledo M, Marmonti E, Orpi M, Capdevila E, Betancourt A, Lopez-Soriano FJ, Argiles JM (2012). Formoterol treatment downregulates the myostatin system in skeletal muscle of cachectic tumour-bearing rats. Oncol Lett 3:185-189.**

**Busquets S, Toledo M, Sirisi S, Orpi M, Serpe R, Coutinho J, Martinez R, Argiles JM, Lopez-Soriano FJ (2011). Formoterol and cancer muscle wasting in rats: Effects on muscle force and total physical activity. Exp Ther Med 2:731-735.**

**Chacon-Cabrera A, Fermoselle C, Salmela I, Yelamos J, Barreiro E (2015). MicroRNA expression and protein acetylation pattern in respiratory and limb muscles of Parp-1(-/-) and Parp-2(-/-) mice with lung cancer cachexia. Biochim Biophys Acta 1850:2530-2543.**

**Chacon-Cabrera A, Fermoselle C, Urtreger AJ, Mateu-Jimenez M, Diament MJ, De Kier Joffe ED, Sandri M, Barreiro E (2014). Pharmacological strategies in lung cancer-induced cachexia: effects on muscle proteolysis, autophagy, structure, and weakness. J Cell Physiol 229:1660-1672.**

**Chacon-Cabrera A, Gea J, Barreiro E (2016a). Short- and Long-Term Hindlimb Immobilization and Reloading: Profile of Epigenetic Events in Gastrocnemius. J Cell Physiol.**

**Chacon-Cabrera A, Lund-Palau H, Gea J, Barreiro E (2016b). Time-Course of Muscle Mass Loss, Damage, and Proteolysis in Gastrocnemius following Unloading and Reloading: Implications in Chronic Diseases. PLoS One 11:e0164951.**

**Fermoselle C, Garcia-Arumi E, Puig-Vilanova E, Andreu AL, Urtreger AJ, De Kier Joffe ED, Tejedor A, Puente-Maestu L, Barreiro E (2013). Mitochondrial dysfunction and therapeutic approaches in respiratory and limb muscles of cancer cachectic mice. Exp Physiol 98:1349-1365.**

**Fermoselle C, Rabinovich R, Ausin P, Puig-Vilanova E, Coronell C, Sanchez F, Roca J, Gea J, Barreiro E (2012). Does oxidative stress modulate limb muscle atrophy in severe COPD patients? Eur Respir J 40:851-862.**

**Fermoselle C, Sanchez F, Barreiro E (2011). [Reduction of muscle mass mediated by myostatin in an experimental model of pulmonary emphysema]. Arch Bronconeumol 47:590-598.**

**Fontes-Oliveira CC, Busquets S, Fuster G, Ametller E, Figueras M, Olivan M, Toledo M, Lopez-Soriano FJ, Qu X, Demuth J, Stevens P, Varbanov A, Wang F, Isfort RJ, Argiles JM (2014). A differential pattern of gene expression in skeletal muscle of tumor-bearing rats reveals dysregulation of excitation-contraction coupling together with additional muscle alterations. Muscle Nerve 49:233-248.**

**Fontes-Oliveira CC, Busquets S, Toledo M, Penna F, Paz AM, Sirisi S, Silva AP, Orpi M, Garcia A, Sette A, Ines GM, Olivan M, Lopez-Soriano FJ, Argiles JM (2013). Mitochondrial and sarcoplasmic reticulum abnormalities in cancer cachexia: altered energetic efficiency? Biochim Biophys Acta 1830:2770-2778.**

**Lopez-Soriano J, Argiles JM, Lopez-Soriano FJ (1997). Sequential changes in lipoprotein lipase activity and lipaemia induced by the Yoshida AH-130 ascites hepatoma in rats. Cancer Lett 116:159-165.**

**Marin-Corral J, Fontes CC, Pascual-Guardia S, Sanchez F, Olivan M, Argiles JM, Busquets S, Lopez-Soriano FJ, Barreiro E (2010). Redox balance and carbonylated proteins in limb and heart muscles of cachectic rats. Antioxid Redox Signal 12:365-380.**

**Marin-Corral J, Minguella J, Ramirez-Sarmiento AL, Hussain SN, Gea J, Barreiro E (2009). Oxidised proteins and superoxide anion production in the diaphragm of severe COPD patients. Eur Respir J 33:1309-1319.**

**Medja F, Allouche S, Frachon P, Jardel C, Malgat M, Mousson de CB, Slama A, Lunardi J, Mazat JP, Lombes A (2009). Development and implementation of standardized respiratory chain spectrophotometric assays for clinical diagnosis. Mitochondrion 9:331-339.**

**Picard B, Barboiron C, Chadeyron D, Jurie C (2011). Protocol for high-resolution electrophoresis separation of myosin heavy chain isoforms in bovine skeletal muscle. Electrophoresis 32:1804-1806.**

**Puig-Vilanova E, Rodriguez DA, Lloreta J, Ausin P, Pascual-Guardia S, Broquetas J, Roca J, Gea J, Barreiro E (2014). Oxidative stress, redox signaling pathways, and autophagy in cachectic muscles of male patients with advanced COPD and lung cancer. Free Radic Biol Med 79C:91-108.**

**Salazar-Degracia A, Blanco D, Vila-Ubach M, de BG, de Solorzano CO, Montuenga LM, Barreiro E (2016). Phenotypic and metabolic features of mouse diaphragm and gastrocnemius muscles in chronic lung carcinogenesis: influence of underlying emphysema. J Transl Med 14:244.**

**Toledo M, Busquets S, Penna F, Zhou X, Marmonti E, Betancourt A, Massa D, Lopez-Soriano FJ, Han HQ, Argiles JM (2016). Complete reversal of muscle wasting in experimental cancer cachexia: Additive effects of activin type II receptor inhibition and beta-2 agonist. Int J Cancer 138:2021-2029.**

**Toledo M, Busquets S, Sirisi S, Serpe R, Orpi M, Coutinho J, Martinez R, Lopez-Soriano FJ, Argiles JM (2011). Cancer cachexia: physical activity and muscle force in tumour-bearing rats. Oncol Rep 25:189-193.**

**Toledo M, Springer J, Busquets S, Tschirner A, Lopez-Soriano FJ, Anker SD, Argiles JM (2014). Formoterol in the treatment of experimental cancer cachexia: effects on heart function. J Cachexia Sarcopenia Muscle 5:315-320.**

**FIGURE LEGENDS**

**Figure S1:** Representative immunoblots of total reactive carbonyls in proteins in the diaphragm (panel A) and gastrocnemius (panel B) muscles of the following experimental groups: non-cachexia controls (N=9), non-cachexia control-F (N=9), cancer-cachexia rats (N=9) and cancer-cachexia-F rats (diaphragm, N=8; and gastrocnemius, N=10). GAPDH is shown as the loading control. Definition of abbreviations: OD, optical densities; a.u., arbitrary units; MW, molecular weight: KDa, kilodaltons; F, formoterol; GAPDH, glyceraldehyde 3-phosphate dehydrogenase.

**Figure S2:** Representative immunoblots of HNE-protein adducts in the diaphragm (panel A) and gastrocnemius (panel B) muscles of the following experimental groups: non-cachexia controls (N=9), non-cachexia control-F (diaphragm, N=9; and gastrocnemius, N=10), cancer-cachexia rats (N=9) and cancer-cachexia-F rats (diaphragm, N=8; and gastrocnemius, N=9). GAPDH is shown as the loading control. Definition of abbreviations: HNE, 4-hydroxy-2-nonenal; OD, optical densities; a.u., arbitrary units; MW, molecular weight: KDa, kilodaltons; F, formoterol; GAPDH, glyceraldehyde 3-phosphate dehydrogenase.

**Figure S3:** Representative immunoblots of MDA-protein adducts in the diaphragm (panel A) and gastrocnemius (panel B) muscles of the following experimental groups: non-cachexia controls (N=9), non-cachexia control-F (N=9), cancer-cachexia rats (diaphragm, N=9; and gastrocnemius, N=10) and cancer-cachexia-F rats (diaphragm, N=8; and gastrocnemius, N=10). GAPDH is shown as the loading control. Definition of abbreviations: MDA, malondialdehyde; OD, optical densities; a.u., arbitrary units; MW, molecular weight: KDa, kilodaltons; F, formoterol; GAPDH, glyceraldehyde 3-phosphate dehydrogenase.

**Figure S4:** Representative immunoblots of total protein tyrosine nitration in the diaphragm (panel A) and gastrocnemius (panel B) muscles of the following experimental groups: non-cachexia controls (N=9), non-cachexia control-F (N=9), cancer-cachexia rats (diaphragm, N=9; and gastrocnemius, N=10) and cancer-cachexia-F rats (diaphragm, N=8; and gastrocnemius, N=10). GAPDH is shown as the loading control. Definition of abbreviations: OD, optical densities; a.u., arbitrary units; MW, molecular weight: KDa, kilodaltons; F, formoterol; GAPDH, glyceraldehyde 3-phosphate dehydrogenase.

**Figure S5:** Representative 2D immunoblots corresponding to the detection of carbonylated proteins in crude muscle homogenates of gastrocnemius of non-cachexia controls (right top panel), non-cachexia controls-F (left top panel), cancer-cachexia rats (right bottom panel) and cancer-cachexia rats (left bottom panel). Isoforms of serotransferina (non-muscle protein) (1), isoforms of pyruvate kinase (2), isoforms of beta-enolase (3), isoforms of acyl-coenzyme A dehydrogenase (4), isoforms of creatine kinase (5), isoforms of fructose biphosphate aldolase A (6), isoforms of glyceraldehyde-3-phosphate dehydrogenase (7), isoforms of carbonic anhydrase-3 (8), isoforms of triose-phosphate isomerase-1 (9), heat shock proteins (non-muscle protein) (10) albumin (non-muscle protein) (11) ATP-synthase (12), actin (13), and tropomyosin (14) were oxidized in the gastrocnemius muscle of the study groups of rats. Definition of abbreviations: MW, molecular weight; kDa, kilodaltons; F, formoterol.

**Figure S6:** Representative immunoblots of SOD1 in the diaphragm (panel A) and gastrocnemius (panel B) muscles of the following experimental groups: non-cachexia controls (N=9), non-cachexia control-F (N=9), cancer-cachexia rats (diaphragm, N=9; and gastrocnemius N=10) and cancer-cachexia-F rats (diaphragm, N=8; and gastrocnemius N=10). GAPDH is shown as the loading control. Definition of abbreviations: SOD1, superoxide dismutase isoform 1; OD, optical densities; a.u., arbitrary units; MW, molecular weight: KDa, kilodaltons; F, formoterol; GAPDH, glyceraldehyde 3-phosphate dehydrogenase.

**Figure S7:** Representative immunoblots of SOD2 in the diaphragm (panel A) and gastrocnemius (panel B) muscles of the following experimental groups: non-cachexia controls (N=9), non-cachexia control-F (N=9), cancer-cachexia rats (diaphragm, N=8; and gastrocnemius, N=10) and cancer-cachexia-F rats (diaphragm, N=9; and gastrocnemius, N=10). GAPDH is shown as the loading control. Definition of abbreviations: SOD2, superoxide dismutase isoform 2; OD, optical densities; a.u., arbitrary units; MW, molecular weight: KDa, kilodaltons; F, formoterol; GAPDH, glyceraldehyde 3-phosphate dehydrogenase.

**Figure S8:** Representative immunoblots of catalase protein in the diaphragm (panel A) and gastrocnemius (panel B) muscles of the following experimental groups: non-cachexia controls (N=9), non-cachexia control-F (N=9), cancer-cachexia rats (N=10) and cancer-cachexia-F rats (N=10). GAPDH is shown as the loading control. Definition of abbreviations: OD, optical densities; a.u., arbitrary units; MW, molecular weight: KDa, kilodaltons; F, formoterol; GAPDH, glyceraldehyde 3-phosphate dehydrogenase.

**Figure S9:** Representative immunoblots of MyHC-I protein in the diaphragm (panel A) and gastrocnemius (panel B) muscles of the following experimental groups: non-cachexia controls (N=9), non-cachexia control-F (N=9), cancer-cachexia rats (N=10) and cancer-cachexia-F rats (N=10). Coomassie blue stained gels were used as the loading control. Definition of abbreviations: MyHC, myosin heavy chain; OD, optical densities; a.u., arbitrary units; MW, molecular weight: KDa, kilodaltons; F, formoterol.

**Figure S10:** Representative immunoblots of MyHC-II protein in the diaphragm (panel A) and gastrocnemius (panel B) muscles of the following experimental groups: non-cachexia controls (N=9), non-cachexia control-F (N=9), cancer-cachexia rats (N=10) and cancer-cachexia-F rats (N=10). Coomassie blue stained gels were used as the loading control. Definition of abbreviations: MyHC, myosin heavy chain; OD, optical densities; a.u., arbitrary units; MW, molecular weight: KDa, kilodaltons; F, formoterol.

**Figure S11:** Representative immunoblots of actin protein in the diaphragm (panel A) and gastrocnemius (panel B) muscles of the following experimental groups: non-cachexia controls (N=9), non-cachexia control-F (N=9), cancer-cachexia rats (diaphragm, N=9; and gastrocnemius, N=10) and cancer-cachexia-F rats (diaphragm, N=8; and gastrocnemius, N=10). GAPDH is shown as the loading control. Definition of abbreviations: OD, optical densities; a.u., arbitrary units; MW, molecular weight: KDa, kilodaltons; F, formoterol; GAPDH, glyceraldehyde 3-phosphate dehydrogenase.

**Figure S12:** Representative immunoblots of creatine kinase protein in the diaphragm (panel A) and gastrocnemius (panel B) muscles of the following experimental groups: non-cachexia controls (N=9), non-cachexia control-F (N=9), cancer-cachexia rats (diaphragm, N=9; and gastrocnemius, N=10) and cancer-cachexia-F rats (diaphragm, N=8; and gastrocnemius, N=10). GAPDH is shown as the loading control. Definition of abbreviations: OD, optical densities; a.u., arbitrary units; MW, molecular weight: KDa, kilodaltons; F, formoterol; GAPDH, glyceraldehyde 3-phosphate dehydrogenase.

**Figure S13:** Representative immunoblots of carbonic anhydrase-3 protein in the diaphragm (panel A) and gastrocnemius (panel B) muscles of the following experimental groups: non-cachexia controls (N=9), non-cachexia control-F (N=9), cancer-cachexia rats (diaphragm, N=9; and gastrocnemius, N=10) and cancer-cachexia-F rats (diaphragm, N=8; and gastrocnemius, N=10). GAPDH is shown as the loading control. Definition of abbreviations: OD, optical densities; a.u., arbitrary units; MW, molecular weight: KDa, kilodaltons; F, formoterol; GAPDH, glyceraldehyde 3-phosphate dehydrogenase.

**
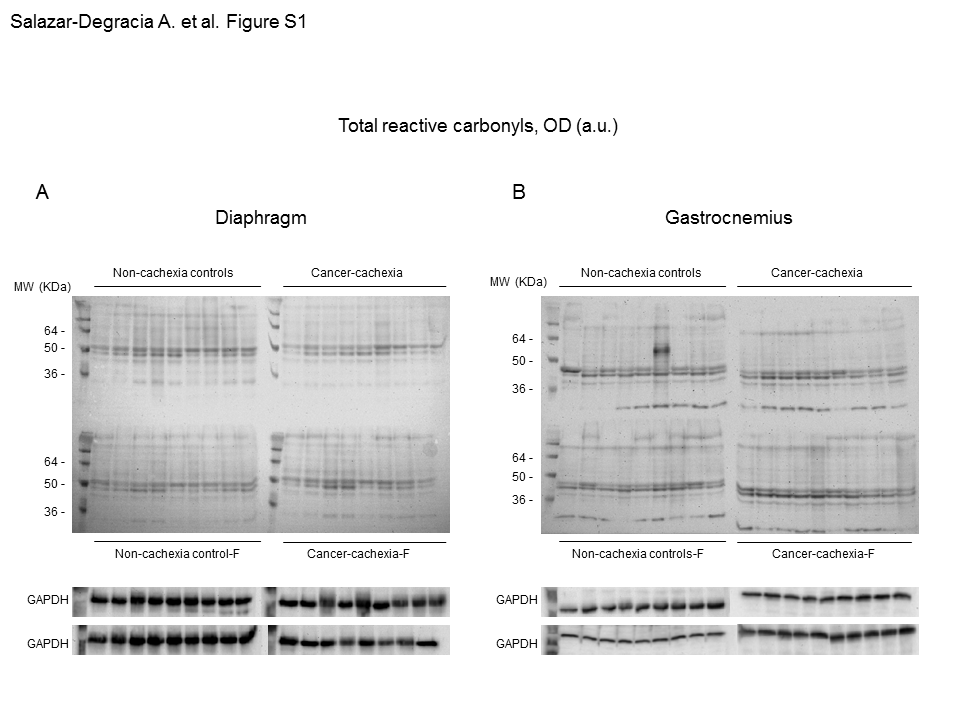

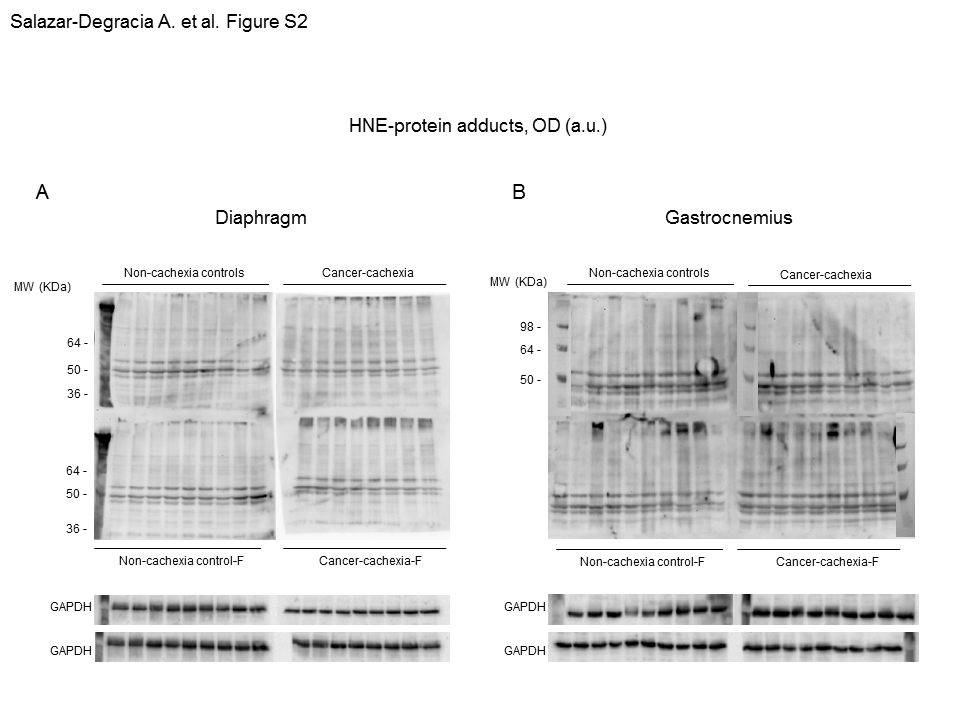

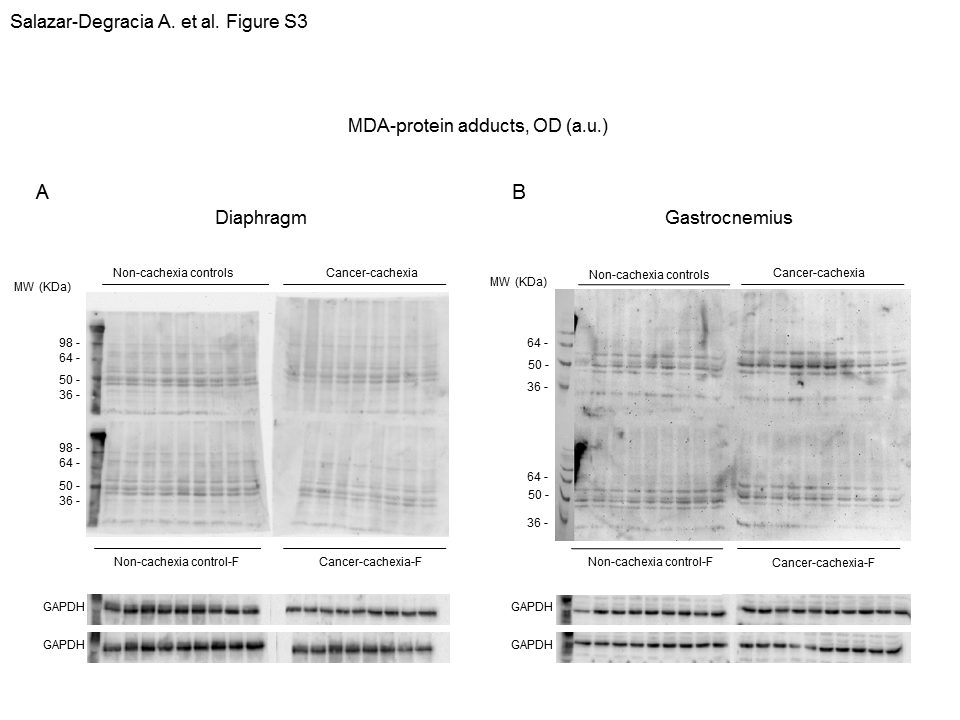

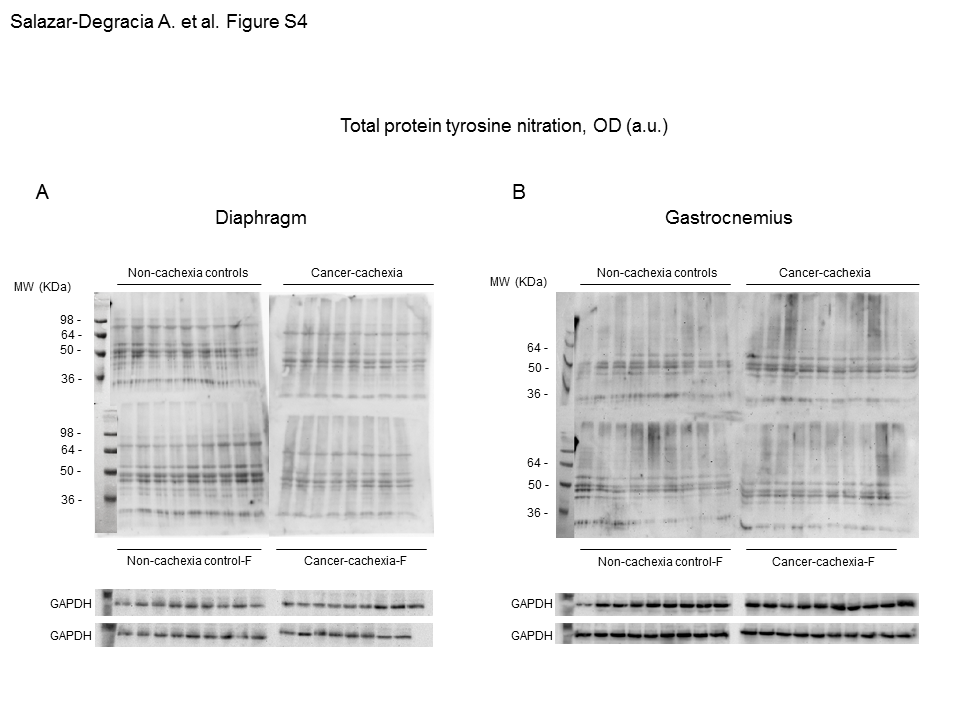

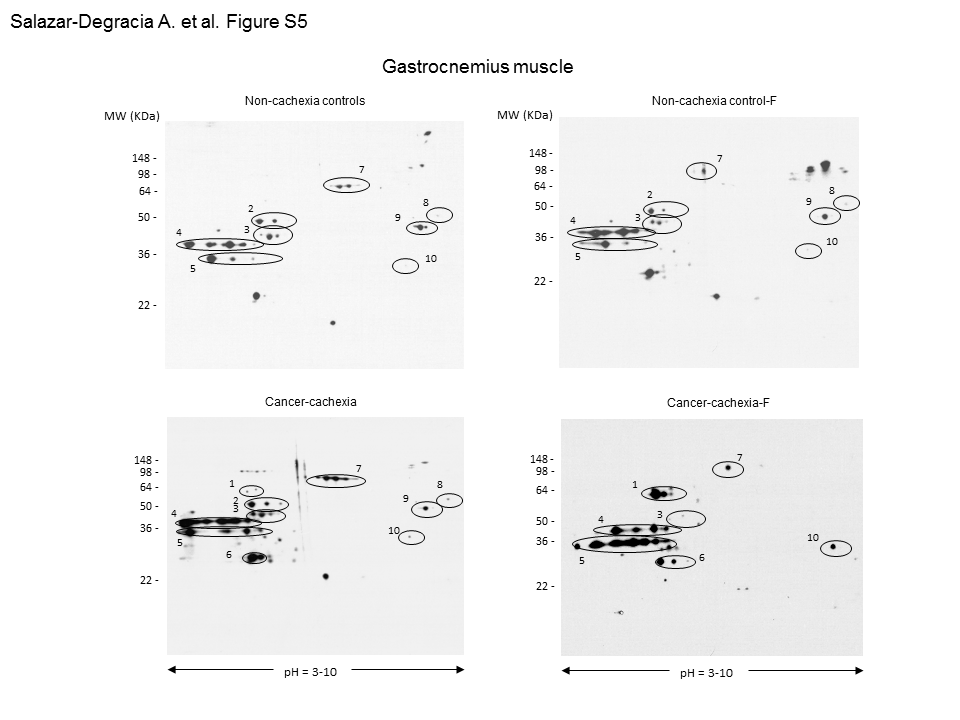

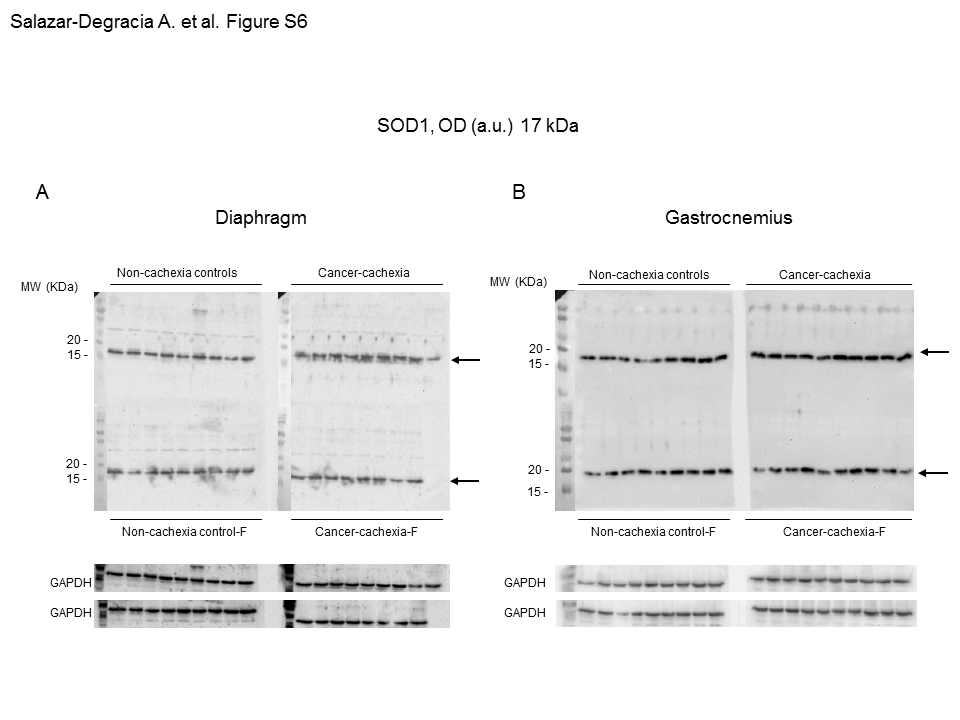

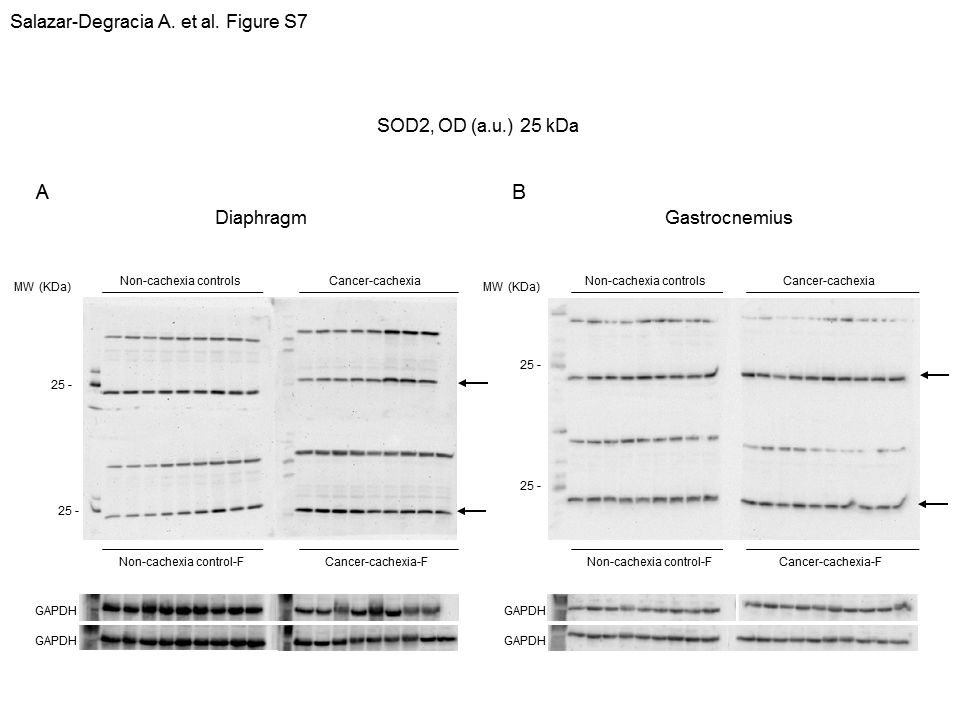

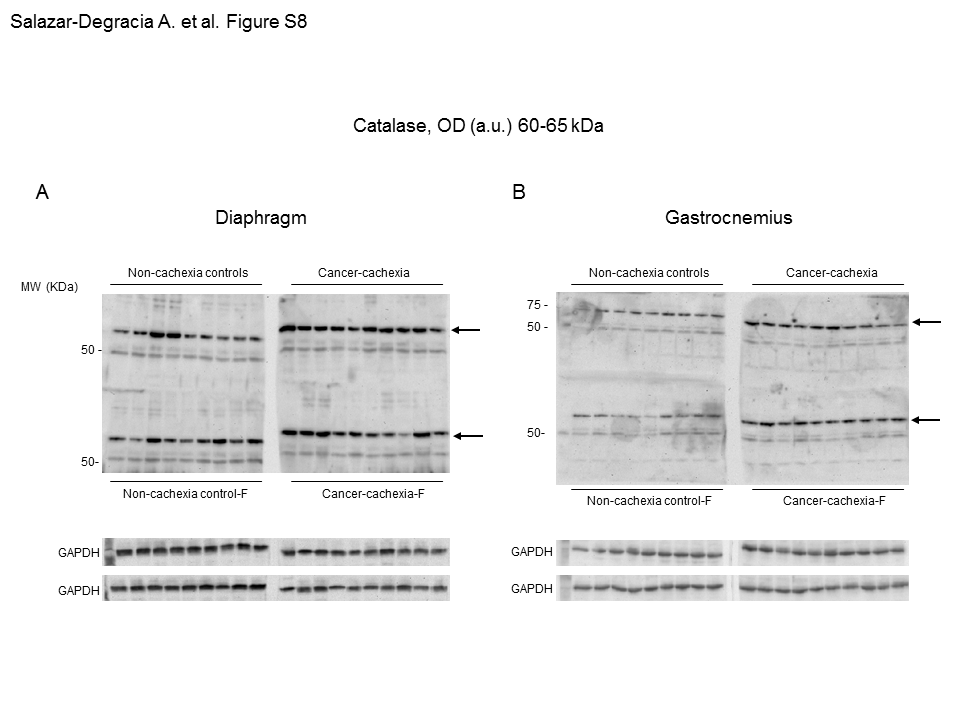

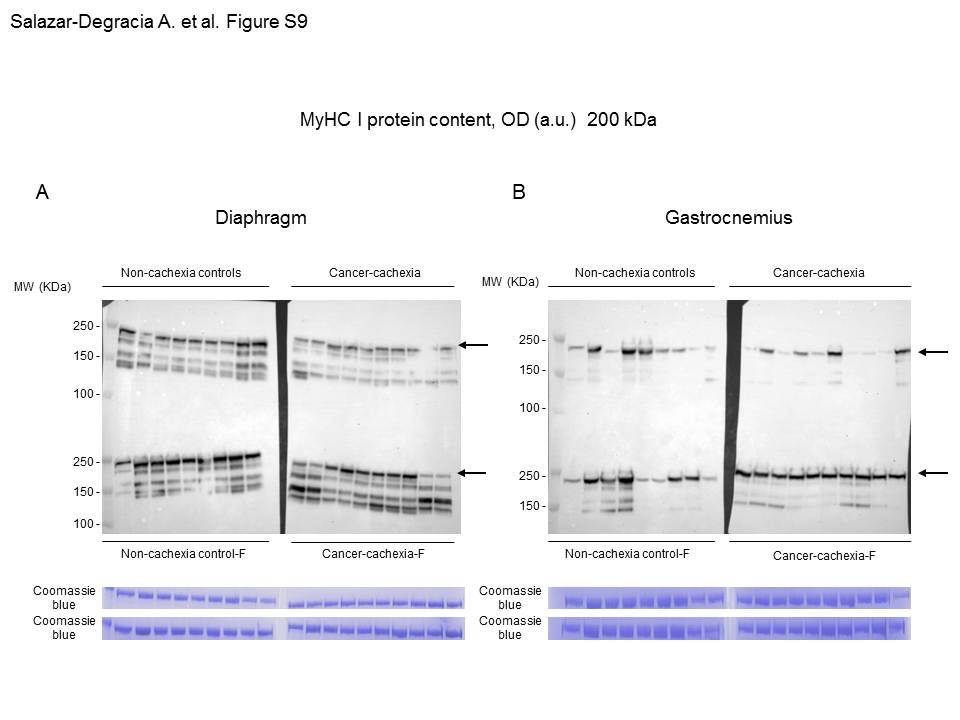

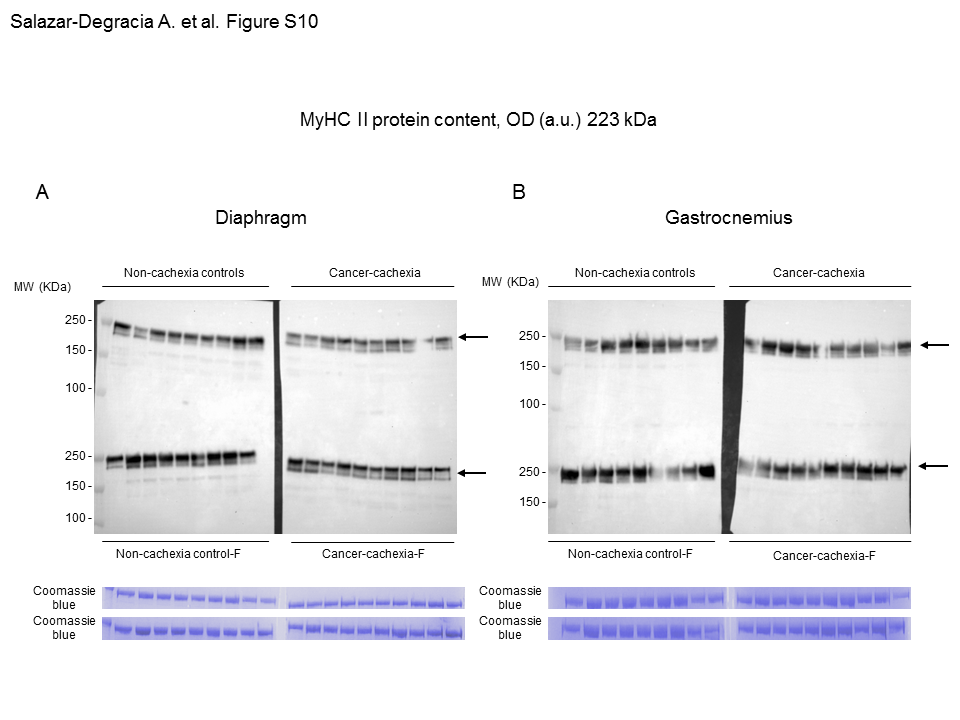

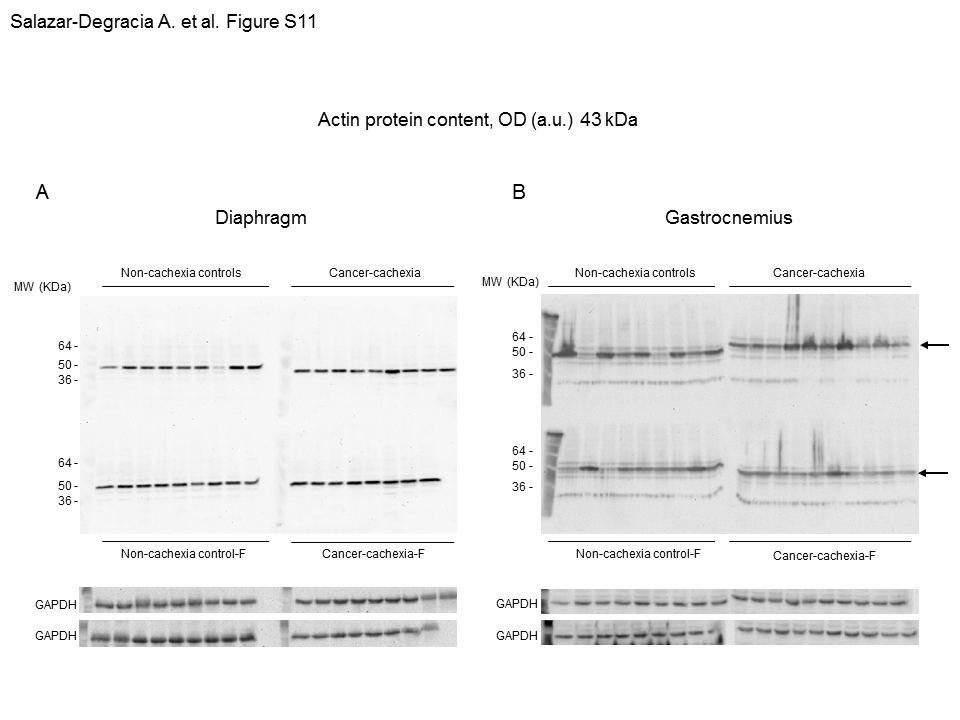

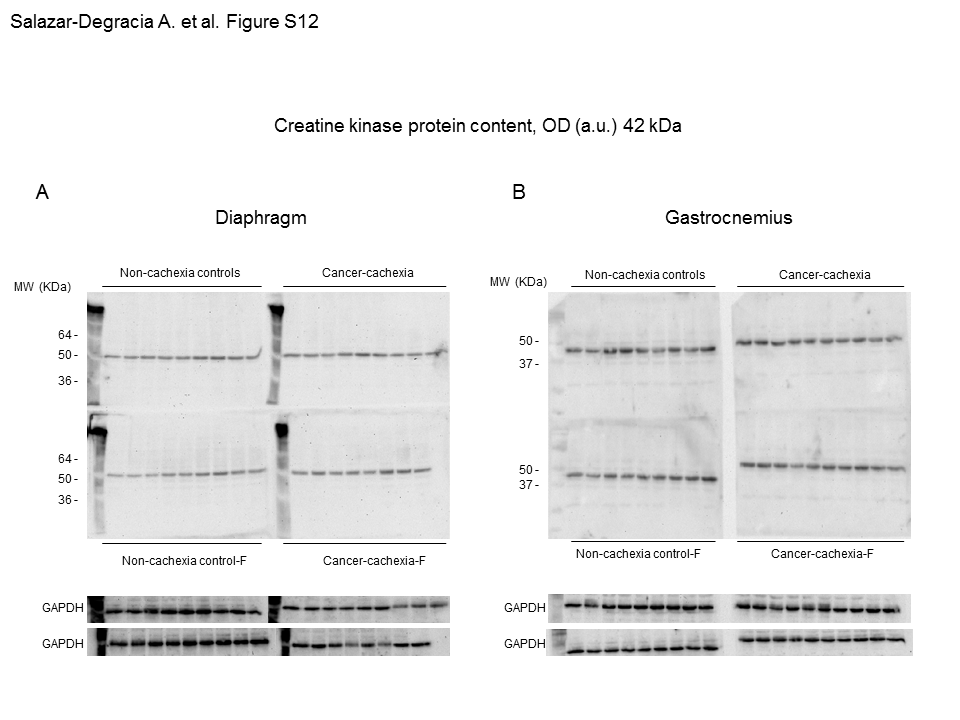

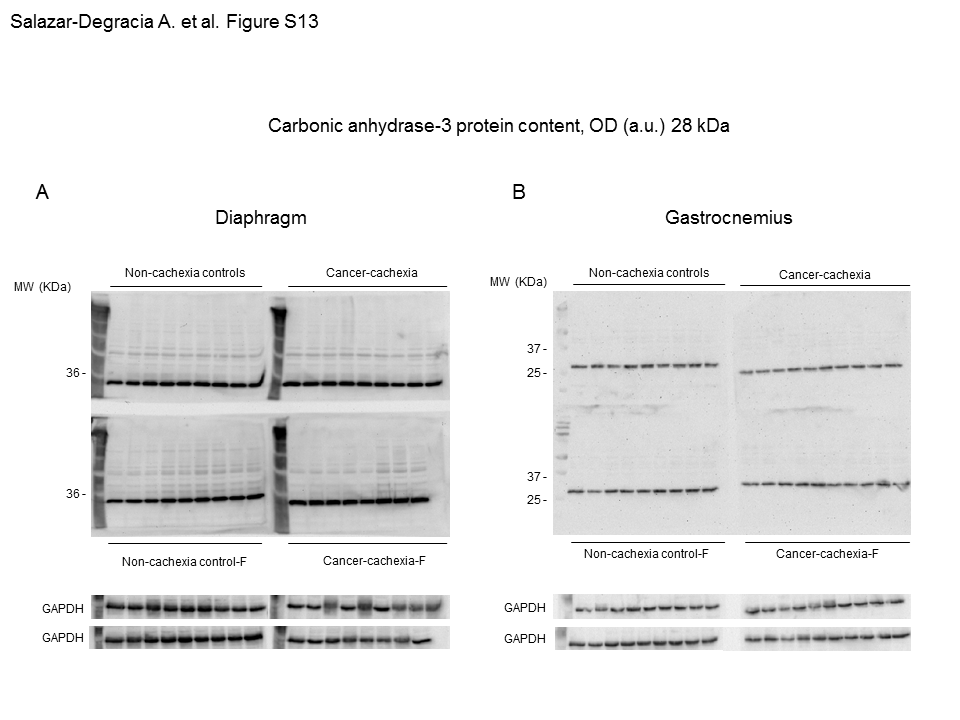
**
